# Identify potent SARS-CoV-2 main protease inhibitors via accelerated free energy perturbation-based virtual screening of existing drugs

**DOI:** 10.1073/pnas.2010470117

**Published:** 2020-10-13

**Authors:** Zhe Li, Xin Li, Yi-You Huang, Yaoxing Wu, Runduo Liu, Lingli Zhou, Yuxi Lin, Deyan Wu, Lei Zhang, Hao Liu, Ximing Xu, Kunqian Yu, Yuxia Zhang, Jun Cui, Chang-Guo Zhan, Xin Wang, Hai-Bin Luo

**Affiliations:** ^a^Guangdong Provincial Key Laboratory of New Drug Design and Evaluation, School of Pharmaceutical Sciences, Sun Yat-Sen University, 510006 Guangzhou, People’s Republic of China;; ^b^Center for Innovative Marine Drug Screening & Evaluation, School of Medicine and Pharmacy, Ocean University of China, 266100 Qingdao, China;; ^c^School of Life Sciences, Lanzhou University, 734000 Lanzhou, China;; ^d^Ministry of Education (MOE) Key Laboratory of Gene Function and Regulation, State Key Laboratory of Biocontrol, School of Life Sciences, Sun Yat-sen University, 510006 Guangzhou, China;; ^e^High Performance Computing Center, Pilot National Laboratory for Marine Science and Technology, 266237 Qingdao, China;; ^f^Marine Biomedical Research Institute of Qingdao, 266100 Qingdao, China;; ^g^State Key Laboratory of Drug Research, Shanghai Institute of Materia Medica, Chinese Academy of Sciences, 201203 Shanghai, China;; ^h^University of Chinese Academy of Sciences, 100049 Beijing, China;; ^i^Guangzhou Institute of Pediatrics, Guangzhou Women and Children’s Medical Center, State Key Laboratory of Respiratory Diseases, Guangzhou Medical University, 510623 Guangzhou, China;; ^j^Molecular Modeling and Biopharmaceutical Center, College of Pharmacy, University of Kentucky, Lexington, KY 40536;; ^k^Department of Pharmaceutical Sciences, College of Pharmacy, University of Kentucky, Lexington, KY 40536;; ^l^Key Laboratory of Tropical Biological Resources of Ministry of Education, School of Life and Pharmaceutical Sciences, Hainan University, 570228 Haikou, China

**Keywords:** virtual screening, SARS-CoV-2, drug repurposing, free energy perturbation, main protease

## Abstract

The COVID-19 pandemic caused by severe acute respiratory syndrome coronavirus 2 (SARS-CoV-2) has become a global crisis. There is no therapeutic treatment specific for COVID-19. It is highly desirable to identify potential antiviral agents against SARS-CoV-2 from existing drugs available for other diseases and thus repurpose them for treatment of COVID-19. In general, a drug repurposing effort for treatment of a new disease, such as COVID-19, usually starts from a virtual screening of existing drugs, followed by experimental validation, but the actual hit rate is generally rather low with traditional computational methods. Here we report a virtual screening approach with accelerated free energy perturbation-based absolute binding free energy (FEP-ABFE) predictions and its use in identifying drugs targeting SARS-CoV-2 main protease (M^pro^). The accurate FEP-ABFE predictions were based on the use of a restraint energy distribution (RED) function, making the practical FEP-ABFE−based virtual screening of the existing drug library possible. As a result, out of 25 drugs predicted, 15 were confirmed as potent inhibitors of SARS-CoV-2 M^pro^. The most potent one is dipyridamole (inhibitory constant K_i_ = 0.04 µM) which has shown promising therapeutic effects in subsequently conducted clinical studies for treatment of patients with COVID-19. Additionally, hydroxychloroquine (K_i_ = 0.36 µM) and chloroquine (K_i_ = 0.56 µM) were also found to potently inhibit SARS-CoV-2 M^pro^. We anticipate that the FEP-ABFE prediction-based virtual screening approach will be useful in many other drug repurposing or discovery efforts.

## Significance

Drug repurposing effort for treatment of a new disease, such as COVID-19, usually starts from a virtual screening of existing drugs, followed by experimental validation, but the actual hit rate is generally rather low with traditional computational methods. It has been demonstrated that a new virtual screening approach with accelerated free energy perturbation-based absolute binding free energy (FEP-ABFE) predictions can reach an unprecedentedly high hit rate, leading to successful identification of 15 potent inhibitors of SARS-CoV-2 main protease (M^pro^) from 25 computationally selected drugs under a threshold of K_i_ = 4 μM. The outcomes of this study are valuable for not only drug repurposing to treat COVID-19 but also demonstrating the promising potential of the FEP-ABFE prediction-based virtual screening approach.

The ongoing pandemic of COVID-19 (1, 2) caused by severe acute respiratory syndrome coronavirus 2 (SARS-CoV-2, also known as 2019-nCoV) has become a global crisis. To date, there is no specific treatment or vaccine for COVID-19. Thus, there is an urgent need to repurpose drugs for treatment of COVID-19 (3). The SARS-CoV-2 replicase gene (Orf1) encodes two overlapping translation products, polyproteins 1a and 1ab (pp1a and pp1ab), which mediate all of the functions required for the viral replication. The main protease (M^pro^) as a key enzyme for the viral replication is initially released by the autocleavage of pp1a and pp1ab. Then, M^pro^ cleaves pp1a and pp1ab to release the functional proteins nsp4 through nsp16 that are necessary for the viral replication (4). In view of the essential functions of M^pro^ in the viral life cycle and its high level of conservation, SARS-CoV-2 M^pro^ is a naturally attractive target for treatment of COVID-19. Hence, there have been efforts to identify therapeutic candidates targeting M^pro^ using various virtual screening methods based on pharmacophore, molecule docking, and molecular simulations (5). As a result of the reported efforts, six drugs were found to inhibit SARS-CoV-2 M^pro^ with a half-maximum inhibitory concentration (IC_50_) ranging from 0.67 μM to 21.4 μM (6). There have been also drug repurposing efforts associated with other potential targets of SARS-CoV-2 (7–9).

In general, a drug repurposing effort for treatment of a new disease, such as COVID-19, usually starts from a virtual screening of existing drugs through computational modeling and simulations, followed by experimental validation. However, the actual hit rate of a virtual screening using traditional computational methods (10, 11) has been rather low, with the vast majority of computationally predicted drug candidates being false positives, because it is difficult to reliably predict protein−ligand binding free energies. Most recently, Gorgulla et al. (12) reported an interesting new virtual screening platform, called VirtualFlow, used to screen numerous compounds in order to identify inhibitors of Kelch-like ECH-associated protein 1 (KEAP1), but the hit rate was still not very high. Within 590 compounds predicted by the virtual screening, 69 were found to be KEAP1 binders (with a hit rate of ∼11.7% for detectable binding affinity), and 10 of these compounds were confirmed to be displacers of nuclear factor erythroid-derived 2-related factor 2 (NRF2) with IC_50_ < 60 μM (with a hit rate of ∼1.4% under the threshold of IC_50_ < 60 μM) (12). Obviously, the hit rate of a virtual screening is dependent on the reliability and accuracy of the receptor−ligand binding free energy predictions used in the virtual screening process. So, the key to the success of a virtual screening effort is use of a reliable computational approach to accurately predict binding free energies.

The free energy perturbation (FEP) simulation of intermolecular interactions (13, 14) is recognized as a reliable method for binding free energy calculations with satisfactory accuracy (13–24), but the traditional FEP method was limited to simulating some minor structural changes of ligands for the relative binding free energy (RBFE) calculations (15, 25). The RBFE calculations can be used to guide lead optimization starting from a promising lead compound (or hit) (15, 25–28), but are not suitable for virtual screening of completely different molecular structures to identify new hits for drug repurposing. For the virtual screening to identify new hits or leads, it is necessary to predict absolute binding free energy (ABFE) for each ligand binding with the target without the requirement to use any reference ligand structure. The FEP-ABFE approach has the advantage of predicting binding affinities between ligands and their targets more accurately than conventional computational methods, such as pharmacophore, molecule docking, and molecular simulations (29–31). However, the previously used FEP-ABFE calculations are extremely expensive and time consuming and, thus, not suitable for virtual screening purposes (that required to screen a large number of compounds) (32, 33).

To make the FEP-ABFE approach practically feasible for our virtual screening and drug repurposing effort, here we report an algorithm using a restraint energy distribution (RED) function to accelerate the FEP-ABFE prediction and its first application to a drug repurposing effort that targets SARS-CoV-2 M^pro^. Our FEP-ABFE prediction-based virtual screening (which predicted 25 drugs as potential inhibitors of SARS-CoV-2 M^pro^) was followed by in vitro activity assays, confirming that 15 out of the 25 drugs can potently inhibit SARS-CoV-2 M^pro^ with 0.04 μM to 3.3 μM (with a remarkably high hit rate of 60% under a threshold of inhibitory constant K_i_ = 4 μM); nine drugs have K_i_ < 1 μM (with a submicromolar hit rate of 36%). Particularly, among these drugs, the most potent inhibitor of SARS-CoV-2 M^pro^ is dipyridamole (DIP, K_i_ = 0.04 μM). Following the computational prediction and in vitro activity validation, DIP was tested for its antiviral activity against SARS-CoV-2 in vitro and in clinical studies for treatment of patients with COVID-19, and the preliminary clinical data are promising for its actual therapeutic effects. While the clinical data are reported separately elsewhere (34), to timely guide further clinical studies and possibly practical clinical application, we describe and discuss in this report the detailed computational and in vitro activity results of DIP along with other promising drugs identified. The encouraging outcomes suggest that the FEP-ABFE prediction-based virtual screening is a truly promising approach to drug repurposing.

## Results and Discussion

### Identification of Potent SARS-CoV-2 M^pro^ Inhibitors for Drug Repurposing.

**Fig. 1.**
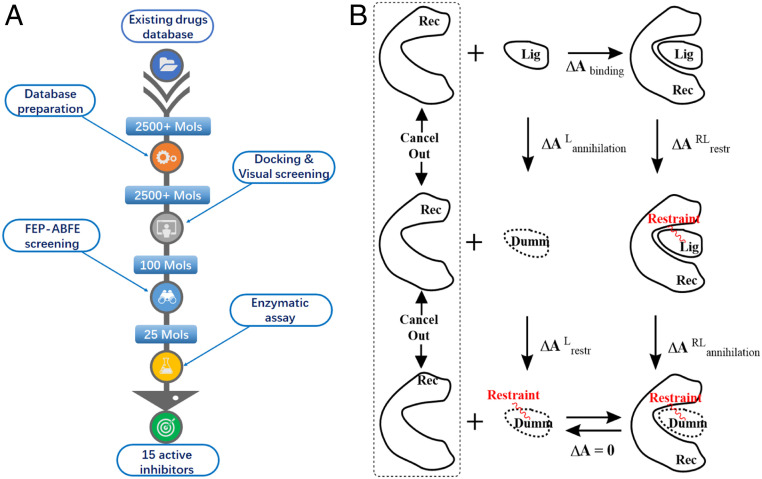
The FEP-ABFE−based screening for the drug repurposing targeting SARS-CoV-2 M^pro^. (*A*) The schedule of FEP-ABFE based screening. (*B*) Thermodynamic cycle used for the FEP-ABFE calculations.

Prior to the virtual screening for drug repurposing, the accuracy of the accelerated FEP-ABFE prediction protocol was validated by using three different protein targets (BRD4, HIV-1 protease, and human factor Xa) and 28 ligands with diverse chemical scaffolds. According to the validation data, given in *SI Appendix*, section S7, the accelerated FEP-ABFE algorithm can achieve a high accuracy for the ABFE predictions. So, in order to identify potent SARS-CoV-2 M^pro^ inhibitors, we first carried out the FEP-ABFE−based virtual screening of all of the existing drugs database, followed by in vitro activity assays, as shown in Fig. 1.

**Fig. 2.**
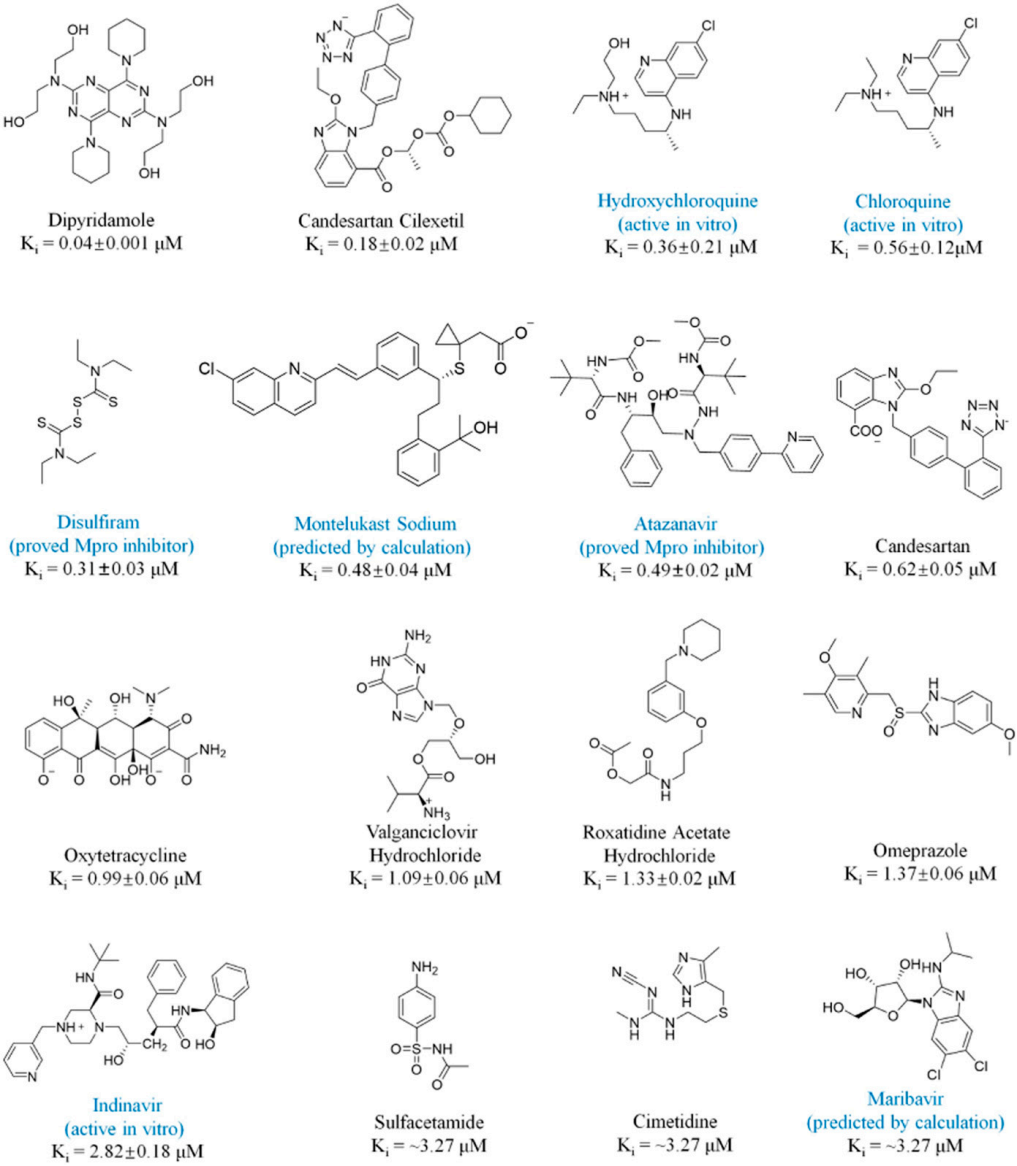
Molecular structures and K_i_ values of 16 confirmed SARS-CoV-2 M^pro^ inhibitors. The seven compounds in blue were also proposed as potential treatments for patients with COVID-19. Within the seven compounds, disulfiram and atazanavir were reported to be SARS-CoV-2 M^pro^ inhibitors with the reported IC_50_ listed in Table 1 (6, 36); hydroxychloroquine, chloroquine, and indinavir were reported to be active in vitro against SARS-CoV-2, but their molecular targets were not reported (9, 37, 39); montelukast sodium and maribavir were only predicted by calculations (37, 38) without experimental activity data reported. Disulfiram and atazanavir served as the positive controls for the in vitro activity [in the literature, the IC_50_ values for disulfiram and atazanavir were 9.35 and 10 μM, respectively (6, 36), and the results of our test were 4.7 and 7.5 μM, respectively, when the same substrate concentration of 20 µM was used].

Specifically, after all of the existing drugs (Dataset S1) were docked into the binding site of SARS-CoV-2 M^pro^, 100 molecules that had specific interactions with the six key amino acid residues, Cys145, His41, Ser144, His163, Gly143, and Gln166, were subjected to further FEP-ABFE calculations. Among these 100 drugs, 49, 46, and 5 were neutral, negatively charged, and positively charged, respectively. Since the FEP method is known to encounter systematic errors when the ligands are not neutral, it is possible that the free energy result calculated for a ligand with +1 or −1 charge may not be compared directly with that calculated for a neutral ligand, due to the possible net charge-caused systematic error. Thus, after the ABFE calculations for all of the 100 ligands were all completed, we grouped the results of the 100 ligands by their net charges. Specifically, all of the 49 neutral ligands are in a group, all of the 5 ligands with +1 charge are in a group, and all of the 46 ligands with −1 charge are in a group. Due to the possible systematic errors between groups, we can only reasonably compare the relative binding free energies for ligands within the same group. Thus, we separately selected the ligands with the highest binding affinities (i.e., the lowest binding free energies) in each group for experimental bioassays. In each group, the top 20 to 40% of the compounds were selected based on their ABFE values. As a result, 25 drugs were selected for subsequent in vitro experimental activity testing. According to the in vitro results, 15 out of these 25 drugs exhibited considerable potency of inhibiting SARS-CoV-2 M^pro^ (Fig. 2 and *SI Appendix*, Fig. S8). DIP, known as an antiplatelet drug which is also a weak inhibitor of microsomal prostaglandin E2 synthase 1 (mPGES-1) (35), was found to be the most potent inhibitor, with K_i_ = 0.04 μM. Following the computational prediction and in vitro activity confirmation, DIP was further tested for its antiviral activity against SARS-CoV-2, demonstrating that DIP dose-dependently suppressed the SARS-CoV-2 replication with a half-maximum effective concentration (EC_50_) of 0.1 μM. The antiviral activity was consistent with the inhibitory activity against M^pro^. In addition, DIP was also tested clinically in treatment of patients with COVID-19, resulting in promising therapeutic data that are reported separately elsewhere (along with the raw antiviral activity data) (34), due to the urgent need of further clinical studies and possibly practical clinical application.

The FEP-ABFE results calculated for all of the confirmed potent SARS-CoV-2 M^pro^ inhibitors are given in Table 1 in comparison with the subsequently determined experimental activity data. As seen in Table 1, 13 out of the 15 FEP-ABFE predicted binding free energies were within 2 kcal/mol of the corresponding experimental values, and, for the other two (disulfiram and maribavir), the deviations were all about 2.2 kcal/mol. Specially for disulfiram, according to its molecular structure, it might be a covalent inhibitor of M^pro^, which could be part of the reason for the relatively larger computational error. However, further studies are needed for disulfiram, to draw a more reliable conclusion. Overall, for the 15 protein−ligand binding complexes, the mean unsigned error (MUE) was about 1.2 kcal/mol. For comparison, we also carried out the molecular mechanics-Poisson–Boltzmann surface area (MM-PBSA) and molecular mechanics-generalized Born surface area (MM-GBSA) calculations on the 15 binding complexes, as given in *SI Appendix*, Table S1, and the MUE values for both of the two methods were larger than 17.0 kcal/mol. Thus, the FEP-ABFE method is, indeed, much more accurate than both the MM-PBSA and MM-GBSA methods for the drug repurposing prediction.

**Table 1.** Summary of the FEP-ABFE calculation results (in kcal/mol) for the experimentally confirmed SARS-CoV-2 M^pro^ inhibitors

Name	IC_50_ (µM)*	K_i_ (µM)^†^	ΔG_exp_	ΔG_FEP-ABFE_	UE_FEP-ABFE_^‡^
DIP	0.60 ± 0.01	0.04 ± 0.001	−10.1	−8.6 ± 0.2	1.5
Candesartan cilexetil	2.8 ± 0.3	0.18 ± 0.02	−9.2	−8.6 ± 0.4	0.6
Hydroxychloroquine	2.9 ± 0.3	0.36 ± 0.21^§^	−8.7	−9.8 ± 0.2	1.1
Chloroquine	3.9 ± 0.2	0.56 ± 0.12^§^	−8.5	−10.0 ± 0.2	1.5
Disulfiram	4.7 ± 0.4 (9.35 ± 0.18)^¶^	0.31 ± 0.03	−8.8	−6.6 ± 0.1	2.2
Montelukast sodium	7.3 ± 0.5	0.48 ± 0.04	−8.6	−7.5 ± 0.4	1.1
Atazanavir	7.5 ± 0.3 (10)^¶^	0.49 ± 0.02	−8.6	−8.0 ± 0.4	0.6
Oxytetracycline	15.2 ± 0.9	0.99 ± 0.06	−8.2	−8.8 ± 0.4	0.6
Valacyclovir hydrochloride	16.7 ± 0.9	1.09 ± 0.06	−8.1	−6.2 ± 0.2	1.9
Roxatidine acetate hydrochloride	20.3 ± 0.4	1.33 ± 0.02	−8.0	−7.2 ± 0.2	0.8
Omeprazole	21.0 ± 1.0	1.37 ± 0.06	−8.0	−6.4 ± 0.2	1.6
Indinavir	43.1 ± 2.8	2.82 ± 0.18	−7.6	−8.0 ± 0.4	0.4
Sulfacetamide	∼50^#^	∼3.27	−7.5	−7.0 ± 0.1	0.5
Cimetidine	∼50^#^	∼3.27	−7.5	−6.1 ± 0.5	1.4
Maribavir	∼50^#^	∼3.27	−7.5	−5.3 ± 0.2	2.2
MUE					1.2

The unsigned error (UE) and MUE values are also given. ΔG_exp_ values were calculated from their corresponding K_i_ values.

* IC_50_ values when the substrate concentration was 20 µM.

^†^ K_i_ values for other molecules were converted from IC_50_ based on the assumption of the competitive inhibition without covalent binding.

^‡^ UE_FEP-ABFE_ = |ΔG_FEP-ABFE_ − ΔG_exp_|.

**Fig. 3.**
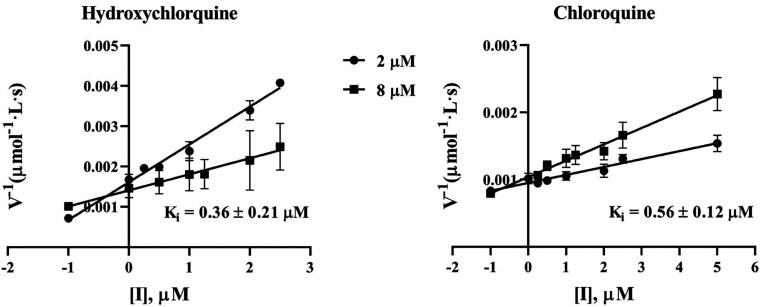
Chloroquine and hydroxychloroquine were identified as SARS-CoV-2 M^pro^ inhibitors with K_i_ = 0.56 and 0.36 μM, respectively. K_i_ was determined according to the enzymatic kinetics using the Dixon plots.

^§^ K_i_ values for hydroxychloroquine and chloroquine were determined using the Dixon plots using the data in Fig. 3.

^¶^ IC_50_ values in the brackets are obtained from other published works, and the published values (6, 36) are close to our experiment results.

^#^ Estimated based on the single-concentration assay showing that the compound at 50 µM inhibited SARS-CoV-2 M^pro^ for over 50%.

Notably, candesartan cilexetil with K_i_ = 0.18 μM against SARS-CoV-2 M^pro^ is a prodrug for its labeled use (treatment of hypertension and congestive heart failure). Hence, we also computationally and experimentally examined its metabolite, candesartan (the active drug corresponding to the prodrug for the labeled use), which was not in the drug library screened. Interestingly, candesartan was also confirmed as a potent inhibitor of SARS-CoV-2 M^pro^, with a slightly lower inhibitory activity against SARS-CoV-2 M^pro^ (K_i_ = 0.62 μM).

Altogether, a total of 16 potent inhibitors of SARS-CoV-2 M^pro^ were identified in this study, and their molecular structures and in vitro inhibitory activity data are shown in Fig. 2 and *SI Appendix*, Fig. S8. Among these 16 compounds, nine (with names shown in black in Fig. 2) were identified as potential candidate treatments of patients with COVID-19, in this study. The remaining seven drugs, including hydroxychloroquine, chloroquine, disulfiram, montelukast sodium, atazanavir, indinavir, and maribavir, were also proposed as potential candidate treatments for patients with COVID-19 in previous studies (6, 9, 36–38). However, within these seven drugs, only disulfiram and atazanavir were previously identified as SARS-CoV-2 M^pro^ inhibitors, whereas the other five drugs were either reported to be active in vitro against SARS-CoV-2 without knowing the specific targets or predicted by computational modeling only without knowing their actual experimental activity. All these drugs were confirmed to be potent SARS-CoV-2 M^pro^ inhibitors in this study. Overall, a total of 14 compounds were confirmed as potent SARS-CoV-2 M^pro^ inhibitors in this study.

Within the SARS-CoV-2 M^pro^ inhibitors identified, DIP is the most potent one, with K_i_ = 0.04 μM (or 40 nM). The computationally modeled structure of DIP binding with SARS-CoV-2 is depicted in *SI Appendix*, Fig. S9 (showing the roles of key residues of the protease, including Thr25, Asn142, Gly143, Ser144, His163, and Glu166, for binding with DIP).

### Molecular Mechanism for the Antiviral Activity of Chloroquine and Hydroxychloroquine Against SARS-CoV-2.

Notably, chloroquine and hydroxychloroquine are currently under clinical trials for treatment of patients with COVID-19, although the exact molecular mechanism and drug target(s) have not been confirmed. Concerning the molecular mechanism for their known antiviral activity, chloroquine or hydroxychloroquine was previously proposed to inhibit acidification of endosome and viral endocytosis (40, 41). However, vesicular stomatitis virus (VSV), which serves as a model virus belonging to *Rhabdoviridae* and has a similar endocytosis process as coronavirus, was not as sensitive as SARS-CoV-2 to hydroxychloroquine and chloroquine (*SI Appendix*, Fig. S10); no significant inhibition was observed for hydroxychloroquine or chloroquine at a concentration of 6.25 μM. Compared to VSV, coronavirus is much more sensitive to chloroquine and hydroxychloroquine. Hydroxychloroquine inhibited SARS-CoV-2 at EC_50_ of 0.72 μM, and chloroquine reduced SARS-CoV replication to 53% at 1.0 μM (42). We wondered whether chloroquine and its analog hydroxychloroquine would directly target a viral protein of coronavirus. In this study, we demonstrated that chloroquine and its analogs inhibited the main protease (M^pro^) activity, which is an essential and conserved enzyme in *Coronaviridae*. Chloroquine and hydroxychloroquine are potent inhibitors of SARS-CoV-2 M^pro^, with K_i_ = 0.56 and 0.36 μM, respectively (Fig. 3). Hence, we cautiously concluded that chloroquine and hydroxychloroquine prevented SARS-CoV-2 infection by inhibition of M^pro^ in addition to the well-known mechanism of abrogation of viral endocytosis. Moreover, norovirus, which belongs to *Caliciviridae* and encodes a viral 3C-like protein similar to M^pro^ of coronavirus, was hypersensitive to chloroquine treatment (43), further suggesting that chloroquine and its analogs may inhibit viral 3C-like protease and inhibit viral replication. The K_i_ value of 0.36 μM for hydroxychloroquine against SARS-CoV-2 M^pro^ is slightly lower than the reported EC_50_ of 0.72 μM against SARS-CoV-2 (42), which is consistent with the possible molecular mechanism that the antiviral activity of hydroxychloroquine against SARS-CoV-2 is mainly due to the inhibitory activity against SARS-CoV-2 M^pro^. The discrepancy between the K_i_ and EC_50_ values may be attributed to the possibly imperfect intracellular drug bioavailability such that the intracellular drug concentration is different from the externally added drug concentration.

Overall, hydroxychloroquine or chloroquine is expected to have both some beneficial effect associated with its antiviral activity due to the SARS-CoV-2 M^pro^ inhibition and adverse side effects associated with other complicated mechanisms of the drug. For example, both chloroquine and hydroxychloroquine would induce the QT interval (the time from the start of the Q wave to the end of the T wave on an electrocardiogram) by blocking human ether-a-go-go−related gene (hERG) encoded potassium channel Kv11.1 [IC_50_ = 2.5 and 10.7 μM, respectively (44–46); the two IC_50_ values are close to their inhibitory potency against M^pro^]. It is also known that chloroquine and hydroxychloroquine inhibit viral endocytosis by raising the pH of endosome/lysosome which would interfere the endocytic membrane transportation of host cells (40). In addition, compared to their distributions in the plasma and other cells susceptible to SARS-CoV-2 infection, chloroquine and hydroxychloroquine distribute more extensively into red blood cells, with a whole blood to plasma ratio of ∼3.8 (47), that are critical for oxygen transportation. Hence, chloroquine and hydroxychloroquine may also negatively affect the oxygen transportation.

Further, in light of our finding that these drugs are potent SARS-CoV-2 M^pro^ inhibitors, it would be interesting to design hydroxychloroquine analogs that can more potently and selectively inhibit SARS-CoV-2 M^pro^ without the unwanted adverse effects of hydroxychloroquine. Similar drug development strategies may also apply to development of analogs of other confirmed SARS-CoV-2 M^pro^ inhibitors such as DIP and candesartan cilexetil with further improved potency and selectivity for SARS-CoV-2 M^pro^.

## Conclusion

By using the accelerated FEP-ABFE predictions for drug repurposing targeting SARS-CoV-2 M^pro^, followed by experimental validation, we successfully identified a total of 16 potent inhibitors of SARS-CoV-2 M^pro^ from existing drugs, including 14 SARS-CoV-2 M^pro^ inhibitors that were confirmed (with K_i_ = 0.04 µM to 3.3 µM) in this study. The identified most potent SARS-CoV-2 M^pro^ inhibitor is DIP (with K_i_ = 0.04 µM) which is currently under clinical studies for treatment of patients with COVID-19, with the promising therapeutic effects reported in a separate report (34). Among other newly identified SARS-CoV-2 M^pro^ inhibitors, prodrug candesartan cilexetil and the corresponding drug candesartan both can potently inhibit SARS-CoV-2 M^pro^.

Additionally, hydroxychloroquine (K_i_ = 0.36 µM) and chloroquine (K_i_ = 0.56 µM) were found to potently inhibit SARS-CoV-2 M^pro^ in this study, suggesting that the previously known antiviral activity of hydroxychloroquine or chloroquine might be mainly due to the inhibitory activity against SARS-CoV-2 M^pro^, in addition to other well-known mechanisms. Further, based on the finding that these drugs are potent SARS-CoV-2 M^pro^ inhibitors, it would be interesting to design hydroxychloroquine analogs that can more potently and selectively inhibit SARS-CoV-2 M^pro^ to improve its antiviral activity and avoid the unwanted adverse effects of hydroxychloroquine associated with other mechanisms. Similarly, the identified other drugs, such as DIP and candesartan cilexetil etc., can also be used as promising starting drug structures to design new drug candidates with further improved potency and selectivity for SARS-CoV-2 M^pro^.

In summary, the virtual screening through accelerated FEP-ABFE predictions has demonstrated an excellent accuracy, with a remarkably high hit rate of 60% under a threshold of K_i_ = 4 μM. We anticipate that the FEP-ABFE prediction-based virtual screening approach will be useful in many other drug repurposing or discovery efforts.

## Methods

### Virtual Screening Based on Accelerated FEP-ABFE Approach.

The accelerated FEP-ABFE approach was based on the use of a RED function. The RED function was derived to automatically add restraints that allow us to perform single-step perturbation (with λ directly from 0 to 1) for accurate binding free energy predictions and, thus, accelerate the FEP-ABFE calculations. The accelerated FEP-ABFE approach is extensively tested and evaluated; see details in *SI Appendix*, sections S1–S7. Briefly, compared to the previously reported FEP-ABFE approaches which normally use 42 λ values (32, 33), the RED function-based FEP-ABFE can be calculated by using just 16 λ values. With such acceleration, the application of FEP-ABFE calculations in virtual screening was made possible. The accuracy of the accelerated 16-λ FEP-ABFE calculation was then tested against 28 ligands with diverse chemical scaffolds, as given in *SI Appendix*, section S7. The test results suggested that the accelerated FEP-ABFE algorithm can achieve a remarkable accuracy, which encouraged us to perform the FEP-ABFE prediction-based practical virtual screening to identify SARS-CoV-2 M^pro^ inhibitors for drug repurposing.

During the virtual screening, molecular docking was first performed by using the crystal structure (Protein Data Bank ID code 6LU7) (6) of SARS-CoV-2 M^pro^ which causes COVID-19. More than 2,500 small compounds in the existing drug library (including all Food and Drug Administration-approved drugs) were screened first by the molecular docking method, and the top 100 ligands were selected by molecular docking and further evaluated by the accelerated FEP-ABFE calculations. Compounds with the lowest binding free energies for each group were selected for further in vitro activity assays. The detailed method for FEP-ABFE−based virtual screening is given in *SI Appendix*, section S1. The derivation of the RED function and extensive evaluations of the accelerated FEP-ABFE method are given, in detail, in *SI Appendix*, sections S2–S7.

### In Vitro Activity Assays of the SARS-CoV-2 M^pro^ Inhibitors.

The pGEX4T1-M^pro^ plasmid was constructed (AtaGenix) and transfected into the *Escherichia coli* strain BL21 (CodonPlus, Stratagene). A GST-tagged protein was purified by GST-glutathione affinity chromatography and cleaved with thrombin. The purity of the recombinant protein was greater than 95% as assessed by sodium dodecyl sulfate polyacrylamide gel electrophoresis (*SI Appendix*, Fig. S11). The catalytic activity of M^pro^ was measured by continuous kinetic assays, using an identical fluorogenic substrate MCA-AVLQSGFR-Lys (Dnp)-Lys-NH2 (Apetide Co., Ltd). The fluorescence intensity was monitored with a Multifunctional Enzyme Marker (SpectraMaxi3x, Molecular Devices) using wavelengths of 320 and 405 nm for excitation and emission, respectively. The experiments were performed in a 100-μL reaction system with a buffer consisting of 50 mM Tris⋅HCl (pH 7.3), 1 mM (ethylenedinitrilo)tetraacetic acid. We first detected the SARS-CoV-2 M^pro^ catalytic efficiency as described previously, with minor modifications (6). In brief, the catalytic efficiency (k_cat_/K_m_, i.e., the ratio of the catalytic rate constant to the Michaelis–Menten constant) of M^pro^ was determined as 25,600 M^−1^⋅s^−1^, which is similar to the previously reported value (k_cat_/K_m_ = 28,500 M^−1^⋅s^−1^) (6). Experiments were performed by mixing 96 nM M^pro^ with different concentrations of substrate (0.03 to ∼2 μM), and the K_m_ (Michaelis–Menten constant) and V_max_ (maximum velocity of the enzymatic reaction) values were calculated from a double-reciprocal plot. To measure the IC_50_ of a compound, 500 nM of enzyme, 20 μM of substrate, and the compound at six different concentrations were added into different wells. The compound was dissolved and diluted in dimethyl sulfoxide to the desired concentrations. One microliter of diluted compound was added into 50 μL of solution containing 1 μM M^pro^, and then solutions were incubated at room temperature for 10 min. The reaction was initiated by adding 50 μL of substrate. Fluorescence intensity was monitored once every 45 s. Initial reaction velocities were calculated by fitting the linear portion of the curves (within the first 5 min of the progress curves) to a straight line using the program SoftMax Pro and were converted to enzyme activity (substrate cleaved)/second.

## Supplementary Material

Supplementary File

Supplementary File

## Data Availability

Additional computational details and computational and experimental data can be found in *SI Appendix*, sections S1–S7, Figs. S1–S10, and Tables S1–S6. The drug library in sdf format containing 3D structures, names, CAS numbers, and SMILES strings can be found in the drug library file (Dataset S1). The computer code to calculate the free energy change using the RED function can be obtained free of charge from Github (https://github.com/zlisysu/RED-function-alchem).
