# Supplementary material for: Identify potent SARS-CoV-2 main protease inhibitors via accelerated free energy perturbation-based virtual screening of existing drugs
Source: Proc Natl Acad Sci U S A. 2020 Oct 13;117(44):27381–7. doi: 10.1073/pnas.2010470117 (PMC7959488; doi:10.1073/pnas.2010470117)
Supplement: Supplementary File [file pnas.2010470117.sapp.pdf]

Supporting Information

Identify potent SARS-CoV-2 main protease inhibitors via accelerated free energy perturbation-based virtual screening of existing drugs

Zhe Li[1,#], Xin Li[2,3,#], Yi-You Huang[1,#], Yaoxing Wu[4], Runduo Liu[1], Lingli Zhou[4], Yuxi Lin[2,3], Deyan Wu[1], Lei Zhang[4], Hao Liu[5], Ximing Xu[2,7], Kunqian Yu[8,9], Yuxia Zhang[6], Jun Cui[4,*], Chang-Guo Zhan[10,11,*], Xin Wang[2,7,*], and Hai-Bin Luo[1,12,*]

[1]Guangdong Provincial Key Laboratory of New Drug Design and Evaluation, School of Pharmaceutical Sciences, Sun Yat-Sen University, Guangzhou 510006, P. R. China

[2]Center for Innovative Marine Drug Screening & Evaluation (QNLM), School of Medicine and Pharmacy, Ocean University of China, 23 Xianggang E Road, Qingdao, 266100, China

[3]School of Life Sciences, Lanzhou University, 220 Tianshui S Road, Lanzhou, 734000, China

[4] MOE Key Laboratory of Gene Function and Regulation, State Key Laboratory of Biocontrol, School of Life Sciences, Sun Yat-sen University, Guangzhou, 510006, China

[5]High Performance Computing Center, Pilot National Laboratory for Marine Science and Technology (QNLM), 1 Wenhai Road, Aoshanwei, Qingdao, 266237, China

[6]Guangzhou Institute of Pediatrics, Guangzhou Women and Children's Medical Center, State Key Laboratory of Respiratory Diseases, Guangzhou Medical University, Guangzhou, 510623, China

[7]Marine Biomedical Research Institute of Qingdao, Qingdao, 266100, China

[8]State Key Laboratory of Drug Research, Shanghai Institute of Materia Medica, Chinese Academy of Sciences, Shanghai 201203, China

[9]University of Chinese Academy of Sciences, Beijing 100049, China

[10]Molecular Modeling and Biopharmaceutical Center, College of Pharmacy, University of Kentucky, 789 South Limestone Street, Lexington, KY, 40536

[11]Department of Pharmaceutical Sciences, College of Pharmacy, University of Kentucky, 789 South Limestone Street, Lexington, KY, 40536

[12] Key Laboratory of Tropical Biological Resources of Ministry of Education, School of Life and Pharmaceutical Sciences, Hainan University, Haikou 570228, Hainan, China.

Correspondence for submission: Chang-Guo Zhan, 859-323-3943 (TEL); zhan@uky.edu.

www.pnas.org/cgi/doi/10.1073/pnas.2010470117

Table S1. Summary of the FEP-ABFE, MM-PBSA, and MM-GBSA calculation results (in kcal/mol) for the experimentally confirmed SARS-CoV-2 M^{pro} inhibitors. The unsigned error (UE) and mean unsigned error (MUE) values are also given. ΔG_{exp} values were calculated from their corresponding K_i values.

Name	IC_{50} (μM)[d]	K_i (μM)[b]	ΔG_{exp}	$\Delta G_{FEP-ABFE}$	$UE_{FEP-ABFE}$[c]	$\Delta G_{MM-PBSA}$	$UE_{MM-PBSA}$[c]	$\Delta G_{MM-GBSA}$	$UE_{MM-GBSA}$[c]
Dipyridamole (DIP)	0.6±0.01	0.04±0.001	-10.1	-8.6	1.5	-23.7	13.6	-34.5	24.4
Candesartan cilexetil	2.8±0.3	0.18±0.02	-9.2	-8.6	0.6	-36.1	26.9	-38.3	29.1
Hydroxychloroquine	2.9±0.3	0.36±0.21[a]	-8.7	-9.8	1.1	-24.0	14.9	-28.7	19.6
Chloroquine	3.9±0.2	0.56±0.12[a]	-8.5	-10.0	1.5	-24.6	16.1	-33.2	24.7
Disulfiram	4.7±0.4 (9.35±0.18)[e]	0.31±0.03	-8.8	-6.6	2.2	-23.0	14.2	-24.3	15.5
Montelukast sodium	7.3±0.5	0.48±0.04	-8.6	-7.5	1.1	-39.5	30.9	-41.5	32.9
Atazanavir	7.5±0.3 (10)[e]	0.49±0.02	-8.6	-8.0	0.6	-33.0	24.4	-39.2	30.6
Oxytetracycline	15.2±0.9	0.99±0.06	-8.2	-8.8	0.6	-10.0	1.8	-14.6	6.4
Valacyclovir hydrochloride	16.7±0.9	1.09±0.06	-8.1	-6.2	1.9	-20.8	12.7	-18.3	10.2
Roxatidine acetate hydrochlor	20.3±0.4	1.33±0.02	-8.0	-7.2	0.8	-29.2	21.2	-30.5	22.5
Omeprazole	21.0±1.0	1.37±0.06	-8.0	-6.4	1.6	-22.3	14.3	-24.4	16.4
Indinavir	43.1±2.8	2.82±0.18	-7.6	-8.0	0.4	-28.8	21.2	-35.4	27.8
Sulfacetamide	~50	~3.27	-7.5	-7.0	0.5	-14.8	7.3	-13.9	6.4
Cimetidine	~50	~3.27	-7.5	-6.1	1.4	-26.2	18.7	-27.8	20.3
Maribavir	~50	~3.27	-7.5	-5.3	2.2	-25.4	19.5	-31.0	25.7
MUE	-		-	-	1.2	-	17.2	-	20.8

[a] K_i values for hydroxychloroquine and chloroquine were determined using the Dixon plots using the data in Figure 3.
[b] K_i values for other molecules were converted from IC_{50} based on the assumption of the competitive inhibition without covalent binding.
[c] $UE_{FEP-ABFE} = |\Delta G_{FEP-ABFE} - \Delta G_{exp}|$; $UE_{MM-PBSA} = |\Delta G_{MM-PBSA} - \Delta G_{exp}|$; $UE_{MM-GBSA} = |\Delta G_{MM-GBSA} - \Delta G_{exp}|$
[d] IC_{50} values when the substrate concentration was 20 μM.
[e] IC_{50} values in the brackets are obtained from other published works, and the published values are close to our experiment results.(1-3)

Section S1. Detailed method for FEP-ABFE based virtual screening

S1.1 Molecular docking

The crystal structure (PDB ID: 6LU7)(4) of SARS-CoV-2 M^{pro} was used for molecular docking. The protonation states of all the amino-acid residues of the protein were determined by the PROPKA program,(5-8) and the hydrogen-bond networks were further carefully checked. As a result, all histidine residues existed in the neutral state, with histidine 41, 163, 164, and 172 having proton at epsilon position (HIE), and histidine 64 and 80 having proton at delta position (HID). Based on the crystal structure, more than 2500 small molecules in the existing drug library (including all FDA-approved drugs; see sdf file known_drugs_db.sdf provided) were screened first by using the Glide molecular docking program,(9) followed by the accelerated FEP-ABFE calculations (see below). As discussed in other reports,(10) there is a higher probability for the docking program to give a correct binding mode than to give a correct binding affinity. To make sure that the screened molecules are reasonable in their binding with the receptor, the binding modes of all the ligands with the receptor were carefully examined by visual inspection. The structure of M^{pro} was analyzed and the possible key residues were determined. Considering that M^{pro} is a protease, Cys145-His41/Ser144-His163 can act as the nucleophilic agent-acid pair to facilitate the catalytic hydrolysis reaction of the substrate protein, and Gly143 and Gln166 can form hydrogen bonds with the "CO-NH-Cα-CO-NH-Cα" structure of the backbone in the substrate protein. Thus, these 6 residues were considered critical to successful M^{pro} inhibitor screening. After molecular docking, the binding modes of all the ligands were carefully examined, and top-100 molecules with specific interactions with these key residues and relatively favorable docking scores were selected for further accelerated FEP-ABFE simulations to predict their binding free energies with M^{pro}.

S1.2 Free energy perturbation (FEP)

S1.2.1 Preliminary molecular dynamics (MD) simulations. All 100 ligands selected by molecular docking were further evaluated by FEP calculations carried out using the GROMACS-2019 program.(11, 12) Before the FEP calculations, 4-ns preliminary MD simulations were performed for each receptor-ligand complex to improve the fit of the ligand into the binding pocket. All the ligands are parameterized by the general AMBER force field (GAFF).(13) Restrained electrostatic potential (RESP) charge calculations on the ligands were performed using the Gaussian 03 program(14) at the HF/6-31G* level. The parameters of the protein were provided by the AMBER FF14SB force field.(15) The TIP3P force field model(16) was used for water molecules. The systems were neutralized by adding counter ions (Na^+ or Cl^-) whenever necessary. The systems were first energy-minimized by using the steepest descent method for 5000 cycles and then heated from 0 to 298 K in an NVT ensemble for 100 ps. The systems were then equilibrated in an NPT ensemble with a weak restraint of 1000 kJ/mol/nm^2 for 500 ps followed by a 4-ns unconstrained production MD simulation run. The final snapshot of the MD-simulated binding structure was used for the subsequent FEP simulation, and the trajectory of the last 2 ns was analyzed to obtain the parameters for adding restraints between the receptor and ligands.

S1.2.2 Protocol for automatically adding restraints. Based on the preliminary MD simulation results, FEP-ABFE calculations were carried out according to the thermodynamic cycle presented in Figure 1. As shown in the thermodynamic cycle, a restraint is added to the receptor (or Rec, which is M^{pro}) and ligand (or Lig) for each FEP calculation. The strategy of adding restraints, which was proposed first by Boresch *et al.*(17) and used in this study, consists of one distance, two angles, and three dihedral harmonic potentials with a force constant of 10 kcal/mol/Å^2 [rad^2]. The contribution of the restraints to the Lig system (ΔA_{restr}^{L}) was calculated analytically, and the contribution of the restraint to the Rec-Lig system (ΔA_{restr}^{RL}) was calculated by the FEP simulation. According to the strategy, three atoms of the ligand and three atoms of the receptor are selected and added to the restraints. To add the restraints at the equilibrium position, an in-house program was coded to automatically detect the required parameters and select the three ligand atoms and the three receptor atoms. For the ligand, the heavy atom closest to the

geometric center is selected as the first one; the heavy atom that is most distant from the first atom is selected as the second one; the heavy atom which forms an angle with the first two atoms larger than 90 degrees and is most distant from the first atom is selected as the third one. For the receptor, based on the last 2-ns trajectory of the 4-ns preliminary MD simulation, the distances, angles, and dihedrals between the three ligand atoms and the C_α, C_c (carbon of the carboxyl group) and O (O atom of the carboxyl group) atoms of all the residues within 5 Å of the ligand were calculated along the MD trajectory. The C_α, C_c, and O atoms from the same residue with the most stable (associated with the lowest standard deviation values) distances, angles, and dihedral values were selected as the three receptor atoms. The mean values for distance, angles, and dihedrals were used for adding the restraints between the three ligand atoms and the three receptor atoms.

S1.3 ABFE calculation

To calculate the ABFE for a ligand with its receptor, the ligand electrostatic and van der Waals interactions are decoupled. In recently published works,(18, 19) 12 λ was used for adding the restraints, 10 λ was used for the decoupling electrostatic interactions, and 20 λ was used for the decoupling vdW interactions, which would be more computationally demanding and, thus, is not suitable for efficient virtual screening.

To make it possible using the FEP-ABFE calculations to rapidly identify M^{pro} inhibitors against COVID-19 from existing drugs, the alchemical pathway was optimized to accelerate the FEP. According to the FEP theory, to calculate the free energy difference ΔA, the probability distribution of the potential energy differences between the adjacent λ, denoted as $P(\Delta U)$, is sampled. In previously reported studies,(20, 21) $P(\Delta U)$ values are considered to have a Gaussian-like distribution, similar to the steps of the electrostatic interaction and vdW interaction decoupling. However, for the added restraint steps, the $P(\Delta U)$ values do not follow a Gaussian distribution. In this study, we first derived and introduced a restraint energy distribution (RED) function which can be used to reasonably describe the $P(\Delta U)$ of the

added restraint steps. By using the automatic restraint-adding program described above and fitting $P(\Delta U)$ values by the RED function, the restraint energy can be estimated accurately by merely using a single-step perturbation (with λ from 0.0 to 1.0), which greatly decreases the computational need necessary for the restraint addition. For the decoupling of electrostatic and vdW interactions, the alchemical pathways that can significantly decrease the number of λ parameters needed to maintain accuracy were also studied. After all these procedures were performed, the FEP-ABFE calculations were accelerated by >3-fold. During the FEP-ABFE calculation on a charged ligand interacting with M^{pro}, annihilation of the charged ligand is associated with a homogeneous compensating background charge change(18, 22), which is an algorithm implemented in the GROMACS-2019 program,(11, 12) in order to ensure that the simulated system is always neutral during the entire FEP simulation process.

After the alchemical pathway was determined, for each window, 5000 cycles of the steepest descent energy minimization were carried out first, and then 100-ps simulations in the NVT ensemble along with Langevin dynamics(23, 24) for temperature coupling were performed to heat the system to 298 K with weak position restraints of 1000 kJ/mol/nm^2 applied to the receptor and the ligand heavy atoms. The simulation system was subsequently equilibrated in an NPT ensemble for 500 ps with the position restraints continuously applied, followed by 4-ns unconstrained production MD simulation. Pressure was coupled using the Parrinello–Rahman pressure coupling scheme.(25) The LINCS constraint algorithm(26) was used only on H-bonds. In all the simulations, the particle mesh Ewald (PME) algorithm(27) was used for the calculation of long-range electrostatic interactions. The ΔU values were sampled during the unconstrained simulation, and the free energy differences between each window were calculated by using the Bennet acceptance ratio (BAR) method.(28, 29)

Several studies(30-32) have indicated that the FEP-ABFE method is relatively accurate for neutral ligands, but when the net charge of the ligand is not 0, a systematic error is encountered.(22) Due to the systematic error related to the net charge of the ligand, the free energy results of the ligand with +1 or -1 charge cannot be directly compared with those of the neutral ligands to determine which one is a more

potent inhibitor. Thus, after we got all the energetic results for the ligands, we grouped the compounds by their net charges, *e.g.* all the neutral ligands as a group, all the ligands with +1 charge as a group, and all the ligands with -1 charge as a group. Within each group, because the ligands have same net charge, there is an error cancellation when comparing their free energy results. The molecules with the lowest binding free energies in each group were selected for further *in vitro* activity assays in order to find more M^{pro} inhibitors regardless of their net charges.

Section S2. Overview of the FEP-ABFE approach using the RED function

The thermodynamic cycle used for the FEP-ABFE calculation is shown in Figure 1 in the main text. The probability distribution of the energy difference between different windows, denoted as $P(\Delta U)$, used for calculating ΔA_{restr}^{RL} can be described by the following restraint energy distribution (RED) function (Eq. S1), with the derivation and detailed discussion of the RED function given in the following section (section S3).

$$P(\Delta U) = \frac{1}{\exp(c\Delta U)^{n_1}} a \cdot b^2 \exp(-b\Delta U) \cdot (\Delta U) + \frac{1}{1 + \left(\frac{d}{\Delta U}\right)^{n_2}} \left(h \frac{1}{\sqrt{\pi}\mu_i} \exp\left(\frac{-(\Delta U - \mu)^2}{2\sigma} \right) \right) \quad (S1)$$

The RED function can accurately describe the sampled restraint energy distribution $P(\Delta U)$ for calculating ΔA_{restr}^{RL}. As shown in Figure S1a, the sampled $P(\Delta U)$ (yellow dots) used for adding restraints can be fitted quite well to the RED function (red line). After fitting the RED function, a series of ΔU values were generated based on the fitted function, and the regenerated ΔU values were then provided to the BAR for the free energy estimation. By using 3 targets and 28 ligands as a test set and the automatic restraint-adding program and then fitting the $P(\Delta U)$ with the RED function, the ΔA_{restr}^{RL} calculated by using single-step perturbation (2 λ states of 0.0 and 1.0) has an excellent correlation with that calculated by using the previously reported method based on 12-λ perturbation with $R^2 > 0.98$ (see Figure S1b). The energy difference between the two alchemical pathways for all the tested systems was less than 0.5 kcal/mol. In addition to using single-step perturbation for the calculation of $\Delta \mathrm{A}_{\mathrm{restr}}^{\mathrm{RL}}$, the

alchemical pathway for calculating $\Delta A_{\text{annihilation}}$ was also optimized, which decreased the number of λ values needed. A detailed discussion of the accuracy of and rationale for using the RED function is provided in SI sections S4 and S5; the detailed results obtained from the single-step perturbation and 12-λ perturbation calculations are compared in SI section S4; and details of the strategy used for further optimizing the alchemical pathway and calculating $\Delta A_{\text{annihilation}}$ are given in SI section S6. The 28 receptor-ligand systems were used to test the FEP-ABFE method, and the method showed remarkable accuracy as discussed in SI section S7. On the basis of all these efforts, the FEP-ABFE can be calculated accurately by using just 16 λ values and, thus, the FEP-ABFE calculation can be accelerated significantly without losing the accuracy of ABFE prediction. The computational acceleration has made the practical FEP-ABFE prediction-based virtual screening for drug repurposing feasible for the first time.

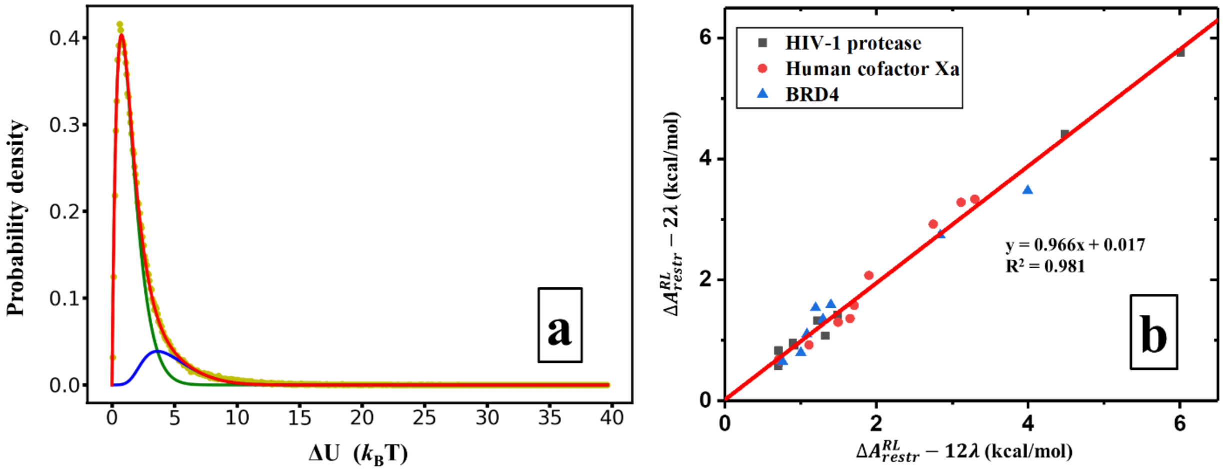

Figure S1. (a) P(Δ*U*) can be fitted well with the RED function. The yellow dots are the sampled P(Δ*U*), the red line is the fitted RED function, the green line is the first term in the RED function, and the blue line is the second term in the RED function. (b) The linear regression of the results of $\Delta A^{\text{RL}}_{\text{restr}}$ calculated using the 12-λ perturbation and using the single-step perturbation methods (denoted as 2 λ), which showed an excellent correlation, with a slope of ~1.0, intercept at ~0.0, and $R^2 > 0.98$.

Section S3. Derivation of the restraint energy distribution (RED) function

Considering the situation where the restraint between the receptor and ligand is added at the equilibrium state (or near-equilibrium state), the interaction between the receptor and ligand consists of two parts including the force field and the restraint. Interactions come from the force field can be simplified as a harmonic potential since the ligand is near the equilibrium, and the restraint force is also a harmonic biasing force. Since the receptor-ligand system is extremely complicated, we will consider a simplified situation where a mass point was originally restrained by a harmonic potential to an equilibrium state (corresponds to the situation that the ligand is restrained by the receptor), and we want to calculate the free energy change for adding an external restraint to the mass center. The initial energy U_{ini} of the simplified system can be represented as

$$U_{ini} = k_{ini}\,(r - r_0)^2 \qquad (S1)$$

where k_{ini} is the initial force constant, and $(r - r_0)$ is the distance between the current position and the equilibrium. When adding restraints, the ΔU between adjacent λ will be sampled in order to calculate the free energy of the added restraints, and the ΔU can be represented by the following equation,

$$\Delta U_{i+1,i} = \Delta\lambda_{i+1,i} k_{res} (r - r_0)^2 \qquad (S2)$$

where i and i+1 means the adjacent ith and $(i + 1)$th window, and k_{res} is the force constant of the added restraint. The energy for the ith window will be

$$U_i = (k_{ini} + \lambda_i k_{res})\,(r - r_0)^2\ . \qquad (S3)$$

Under the above circumstances, the probability distribution of U_i for ith window $P(U_i)$ can be represented as

$$P(U_i) = \frac{exp(-\beta U_i)\Omega(U_i)}{Z} \qquad (S4)$$

where Z is the partition function for ith window, $\beta = (k_B T)^{-1}$, and $\Omega(U_i)$ is the density of state. Since the generalized distance $(r - r_0)$ between the current position (r) and the equilibrium (r_0) can be considered as a three-dimensional vector, the density of state $\Omega(U_i)$ can be represented as

$$\Omega(U_i) = 4\pi(r - r_0)^2. \quad \text{(S5)}$$

By substituting Eqs. (S2), (S3), and (S5) to Eq. (S4), we can get

$$P(U_i) = \frac{exp(-\beta(k_{ini} + \lambda_i k_{res})(r - r_0)^2)4\pi(r - r_0)^2}{Z} \ . \quad \text{(S6)}$$

According to Eqs. (S2) and (S3), both U_i and $(r - r_0)^2$ can be represented by $\Delta U_{i+1,i}$ as the following two equations,

$$(r - r_0)^2 = \Delta U_{i+1,i}/(\Delta\lambda_{i+1,i} k_{res}) \quad \text{(S7)}$$

$$U_i = \Delta U_{i+1,i} \frac{(k_{ini} + \lambda_i k_{res})}{(\Delta\lambda_{i+1,i} k_{res})} \ . \quad \text{(S8)}$$

By substituting (S7) and (S8) to (S6), we have

$$P\left(\Delta U_{i+1,i} \frac{(k_{ini} + \lambda_i k_{res})}{(\Delta\lambda_{i+1,i} k_{res})}\right) = \frac{exp(-\beta \frac{(k_{ini} + \lambda_i k_{res})}{(\Delta\lambda_{i+1,i} k_{res})} \Delta U_{i+1,i}) \frac{4\pi}{(\Delta\lambda_{i+1,i} k_{res})} \Delta U_{i+1,i}}{Z} , \quad \text{(S9)}$$

in which k_{ini}, λ_i, k_{res}, $\Delta\lambda_{i+1,i}$, Z are constant values for a specific state, and the only independent variable in the equation is $\Delta U_{i+1,i}$. After combining the terms which are constant factors, and normalizing the distribution function, $P(\Delta U_{i+1,i})$ has the form of Eq. (S10)

$$P(\Delta U_{i+1,i}) = a \cdot b^2 \exp(-b\Delta U_{i+1,i}) \cdot (\Delta U_{i+1,i}), \quad \text{(S10)}$$

where *a* and *b* are all constants, and b^2 is a factor for normalization purpose. At this point, we get $P(\Delta U_{i+1,i})$ for the idealized model, and we want to know how much this idealized model can reflect the real condition. Thus, Eq. (S10) was fitted to the sampled probability distribution. In the fitting of Eq. (S10), we kept *a* = 1 so that the integral of $P(\Delta U_{i+1,i})$ from 0 to infinity is 1, and only parameter *b* is allowed to change during the fitting, as shown in Figure S2.

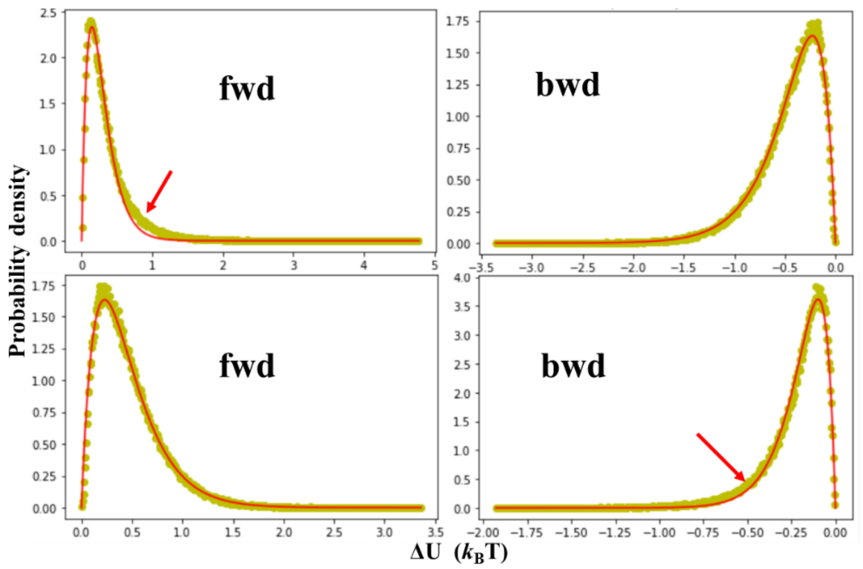

Figure S2. P(Δ*U*) fitted by Eq. (S10). The yellow dots are the sampled P(Δ*U*), the red lines are the fitted function. The ΔU can refer to both the forward and reverse calculations.

We would like to explain why the equation derived from the simplified model can fit the sampled $P(\Delta U)$ well. In the receptor-ligand system, when the ligand comes to an equilibrium at the binding pocket, we can roughly assume that each atom (at least each heavy atom) in the ligand vibrates near its own equilibrium position. The force on each equilibrated atom can be calculated by the force field. We should notice that the force field consists of bond, angle, dihedral, electrostatic, and vdW interactions, and the externally added restraint also consists of one bond, two angles, and three dihedrals interactions. Although the externally added restraint seems contain multiple coordinates and can be quite confusing, the actually adding restraint is just like adding some terms to the originally existed force field. The freedom of each restraint atom does not change, which means that each of the restraint atom can still move in 3-dimensional (3D) space, except that it experiences a stronger restoring force. Near the equilibrium position, each restraint atom is facing the same situation with the atom in the simplified model. Thus, the derived equation S10 can describe the sampled $P(\Delta U)$ so well.

For the points that are far from the equilibrium position, there are some divergence between the equation and the sampled results, as can be seen from the areas pointed out by the red arrows in Figure S2. Eq. (S10) was derived based on a simplified model, in which the original force for the mass point is a harmonic potential. For the receptor-ligand system described by the more complicated force field, the force for an atom that is far from its equilibrium position cannot be approximated by a harmonic potential, which can be the reason for the divergence between the equation and the sampled $P(\Delta U)$. In order to better describe the sampled results, we added a Gaussian term to Eq. (S10) to describe the sampled points that are far from the equilibrium, and the equation can be represented as

$$P(\Delta U) = \frac{1}{\exp(c\Delta U)^{n_1}} a \cdot b^2 \exp(-b\Delta U) \cdot (\Delta U) + \frac{1}{1+\left(\frac{d}{\Delta U}\right)^{n_2}} \left(h \frac{1}{\sqrt{\pi}\mu_i} \exp\left(\frac{-(\Delta U-\mu)^2}{2\sigma}\right)\right) \quad \text{(S11)},$$

in which we represented $\Delta U_{i+1,i}$ as ΔU. Equation (S11) is the restraint energy distribution (RED) function, where a, b, c, d, h, μ, and σ are the parameters to be fitted. In the RED function, $\frac{1}{\exp(c\Delta U)^{n_1}}$ and $\frac{1}{1+\left(\frac{d}{\Delta U}\right)^{n_2}}$ are two factors used to combine the two terms and keep the function to be described only by the first term (denoted as harmonic energy term hereinafter) when ΔU is small and only by the second term (denoted as Gaussian term hereinafter) when ΔU is large. The two constants n_1 and n_2 determine how fast the first term decays and the second term grows, and we eventually chose 4 for both n_1 and n_2.

After the RED function was constructed, the RED function can fit the sampled energy distribution better than Eq. (S10) with no apparent divergences. The results of fitting $P(\Delta U)$ by the RED function is discussed in detail in the following section (SI Section S4).

After fitting the RED function, in theory, the fitted RED function can be used directly for the energy calculation. However, in order to use the existing tools such as pymbar, we can generate a series of ΔU values using the fitted RED function, and the regenerated ΔU values can be provided to the BAR for free energy calculation. Specially, with our implementation, 10,000 bins are selected between the sampled minimum and maximum ΔU values:

```
# code for generating 10000 bins
# self.x is the list for the generated 10000 bins
# u_mean determines whether ΔU values are positive or negative
if self.u_mean > 0:
    # ΔU values are positive, which is the case for forward calculation
    # generate 10000 bins from 0 to maximum ΔU
    self.x = np.linspace(0, 1.0*self.u_l.max(),10000)
else:
    # ΔU values are negative, which is the case for backward calculation
    # generate 10000 bins from minimum ΔU to 0
    self.x = np.linspace(1.0*self.u_l.min(),0, 10000)
# calculate the corresponding probability density.
# self.func_rd is the fitted RED function.
self.fitted_y = self.func_rd(plsq,self.x)
```

Note that for the restraints, the sampled ΔU can only be positive for forward calculation and negative for backward calculation. For each bin of ΔU, we can calculate the corresponding probability density based on the fitted RED function. Based on the probability density and the bin length, we can calculate the probability of the ΔU as P(ΔU) * bin_length. ΔU values are generated with the quantity of 1,000,000 * P(ΔU) * bin_length. The algorithm can easily be implemented in Python by following lines of code:

```
# code for generating ΔU data based on the RED function
# self.fitted_u is the list for newly generated ΔU data, self.x is the 10000 bins
ddu = abs(self.x[2]-self.x[1]) # bin length
self.fitted_u=[]
for i in range(len(self.x)):
    self.fitted_u += [self.x[i] for j in range(round(self.fitted_y[i]*ddu*1000000.0))]
```

This will generate a total of 1,000,000 ΔU values. These regenerated ΔU values are used for further BAR calculations.

Section S4. Based on the aforementioned RED function fitting-based automatic restraint selection, the single-step perturbation calculations with 2 λ states are as accurate as the corresponding calculations with 12 λ states

Although the accuracy of the FEP calculations has been examined by several studies,(18, 19) the computational need for a single FEP-ABFE prediction is massive. Thus, in order to rapidly discover inhibitors for clinical use against COVID-19, it is important to increase the convergence (with respect to the number of λ states) and decrease the computational costs without losing the accuracy of the FEP simulation. Normally, more than 10 λ values will be used during the addition of restraints, such as the works recently reported by Aldeghi *et al.* that 12 non-uniformly distributed λ values (0.0, 0.01, 0.025, 0.05, 0.075, 0.1, 0.15, 0.2, 0.3, 0.5, 0.75, and 1.0) are used for the addition of restraints.(18, 19) However, with the use of the automatic restraint adding program and fitting the probability distribution of the sampled free energy difference $P(\Delta U)$, the $P(\Delta U)$ can be fitted quite well. As a result, the restraint energy can be calculated accurately using just one-step perturbation with λ value changing directly from 0.0 to 1.0, and the calculation can be accelerated greatly. Using a HIV-1 protease-ligand complex (crystal structure PDB ID: 2QHY) as an example, the restraint was added by using single-step perturbation and 12-λ perturbation, respectively. The sampled $P(\Delta U)$'s for adding restraints are fitted by the RED function, and the results are shown in Figure S3. In each picture, the yellow dots are the corresponding sampled $P(\Delta U)$, the green line is the harmonic energy term in the RED function, and the blue line is the Gaussian term in the RED function. As expected, the RED function is described only by the harmonic energy term when ΔU is small and only by the Gaussian term when ΔU is large, and the sampled $P(\Delta U)$ can be fitted well. The free energies of adding restraints are 0.912 and 0.914 kcal/mol for the 12-λ perturbation and the single-step perturbation, respectively, which means the single-step perturbation is sufficient to calculate the restraint energy without negatively impacting accuracy.

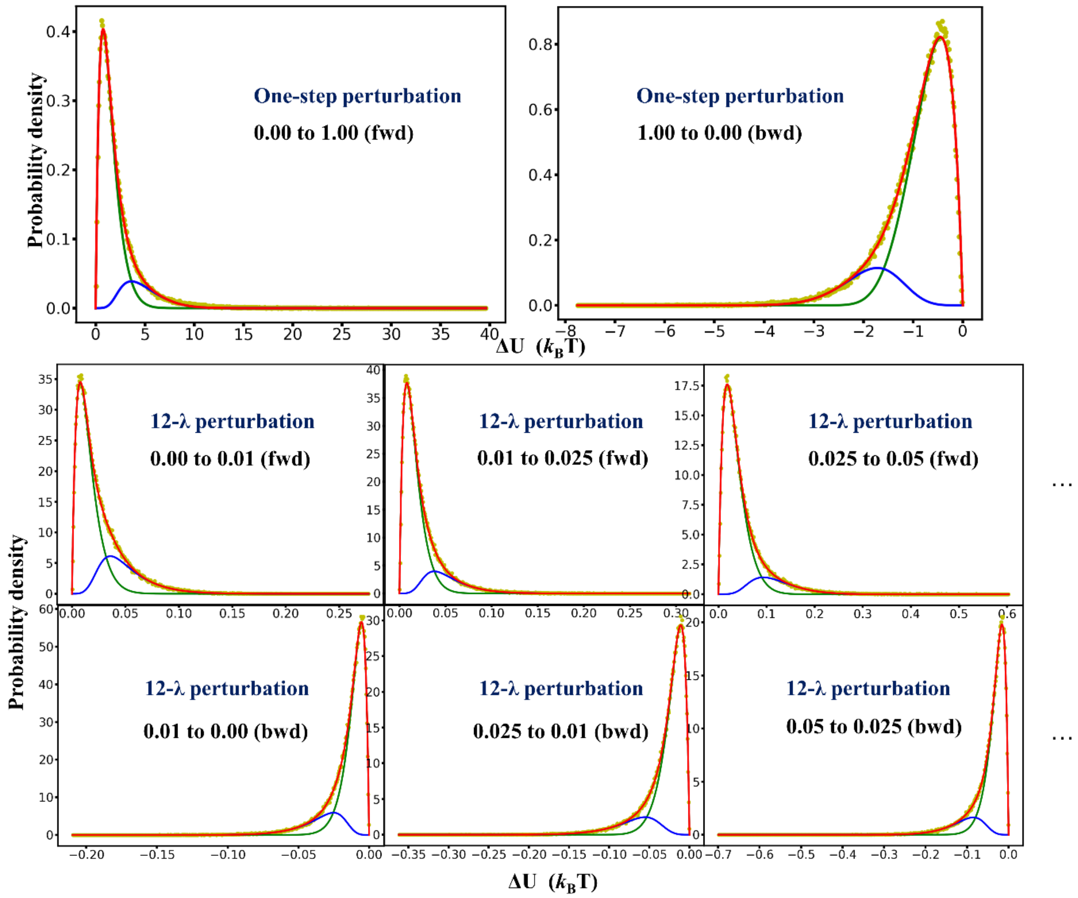

Figure S3. The sampled $P(\Delta U)$'s fitted by the RED function. The yellow dots are the corresponding sampled $P(\Delta U)$, the green line is the harmonic energy term in the RED function, and the blue line is the Gaussian term in the RED function.

In order to further justify the applicability of the single-step perturbation (2 λ with values of 0.0 and 1.0, denoted as 2 λ below) in adding restraints, three different targets and 28 ligands were tested to compare the $\Delta A_{\mathrm{restr}}^{\mathrm{RL}}$ results calculated for the 12 λ and 2 λ states. The bond, angle, and dihedral parameters for the restraints are the same for the calculations with the 12 λ and 2 λ states. Summarized in Table S2 are the $\Delta A_{\mathrm{restr}}^{\mathrm{RL}}$ values calculated using various methods for comparison. According to the results in Table S2, the 2 λ states-based single-step perturbation results ($\Delta A_{\mathrm{2restr\_fit}}^{\mathrm{RL}}$) using the fitted RED function are very close to the corresponding results ($\Delta A_{\mathrm{12restr\_bar}}^{\mathrm{RL}}$) obtained from the calculations with the 12 λ states, with the free energy difference for all the tested systems being less than 0.5 kcal/mol.

In addition, we also tested the BAR calculations using the raw ΔU values obtained from the 2 λ-states sampling, and the obtained results ($\Delta A^{RL}_{2restr\_bar}$) are also listed in Table S2 for comparison. As seen in Table S2, the obtained $\Delta A^{RL}_{2restr\_bar}$ values are also close to the corresponding $\Delta A^{RL}_{2restr\_fit}$ values.

Table S2. Comparison of the ΔA^{RL}_{restr} values (kcal/mol) calculated using various methods.

Protein target	PDB ID	$\Delta A^{RL}_{12restr\_bar}$ [a]	$\Delta A^{RL}_{2restr\_bar}$ [b]	$\Delta A^{RL}_{2restr\_fit}$ [c]	$\Delta\Delta A^{RL}_{restr}$ [d]
HIV-1 protease	2QHY	0.912±0.001	0.940±0.002	0.914±0.001	-0.002
	4U8W	1.486±0.003	1.530±0.004	1.420±0.002	0.066
	5UPZ	1.323±0.002	1.111±0.003	1.075±0.001	0.248
	1AJV	0.708±0.003	0.857±0.002	0.832±0.001	-0.124
	1D4H	4.483±0.005	4.455±0.006	4.410±0.002	0.073
	1D4I	0.897±0.002	0.999±0.002	0.953±0.001	-0.056
	1EBY	1.222±0.002	1.292±0.002	1.324±0.000	-0.102
	1EBZ	0.712±0.003	0.710±0.002	0.685±0.001	0.027
	3A2O	0.706±0.002	0.574±0.001	0.582±0.000	0.124
	1G2K	6.009±0.003	5.951±0.022	5.759±0.012	0.250
Human factor Xa	1F0S	1.900±0.005	2.039±0.007	2.073±0.003	-0.173
	1FJS	1.652±0.006	1.732±0.005	1.361±0.002	0.291
	1MQ6	1.494±0.002	1.333±0.002	1.298±0.001	0.196
	1NFW	3.296±0.005	3.345±0.004	3.334±0.002	-0.038
	1NFX	1.706±0.002	1.776±0.002	1.576±0.001	0.130
	2J34	2.747±0.006	3.106±0.004	2.922±0.002	-0.175
	2P16	0.725±0.002	0.745±0.002	0.656±0.001	0.069
	2P95	0.710±0.002	0.769±0.003	0.684±0.002	0.026
	2XC0	1.111±0.001	0.980±0.002	0.924±0.001	0.187
	2VVV	3.114±0.002	3.479±0.003	3.283±0.002	-0.169
BRD4	3MXF	1.197±0.003	1.244±0.002	1.538±0.001	-0.341
	4MR3	1.398±0.002	1.556±0.002	1.587±0.001	-0.189
	3U5L	1.002±0.003	0.809±0.002	0.797±0.001	0.205
	4MR4	0.761±0.002	0.678±0.002	0.650±0.000	0.111
	3U5J	1.082±0.003	1.355±0.004	1.104±0.001	-0.022
	3SVG	3.992±0.006	3.642±0.006	3.503±0.003	0.489
	4HBV	1.294±0.004	1.288±0.003	1.351±0.001	-0.057
	4J0R	2.839±0.003	2.737±0.004	2.738±0.002	0.101

[a] $\Delta A^{RL}_{12restr\_bar}$ is the restraint energy calculated by using the 12 λ states and BAR method.

[b] $\Delta A^{RL}_{2restr\_bar}$ is the restraint energy calculated from the single-step perturbation using the raw ΔU values from the 2 λ states and the BAR method.

[c] $\Delta A^{RL}_{2restr\_fit}$ is the restraint energy calculated from the single-step perturbation using the fitted RED function to regenerate the ΔU values for the BAR calculations.

[d] $\Delta\Delta A^{RL}_{restr} = \Delta A^{RL}_{12restr\_bar} - \Delta A^{RL}_{2restr\_fit}$

Section S5. The significance of the RED function

During the derivation of the RED function, there was an assumption that the restraint was added exactly at the equilibrium position. If the restraint was added at the equilibrium position, the $P(\Delta U)$ can be described well by the RED function, and the free energy of adding restraint can be calculated by single-step perturbation. During this study, we intentionally tried to add the restraint at the equilibrium position by finding the residue with the most stable distance, angles, and dihedrals with the ligand, and as a result, most of the systems can be described well by the RED function. However, in many cases, especially when the restraint atoms were determined by just inspecting the structure and selecting by experience, there is a higher possibility that the restraints are not added to the equilibrium position, and the sampled ΔU cannot be fitted well by the RED function. For example, in the BRD4-Alprazolam complex, we empirically chose three atoms from both BRD4 and Alprazolam (PDB code: 3U5J), respectively, for adding the restraint. The parameters needed for restraints (including one distance, two angles, and three dihedrals) were also determined according to a 4-ns MD simulation. The atoms for adding the restraint are shown in Figure S4. The only problem with this selection could be that the three atoms on the protein (the side chain of Ile146) may not be very stable, and thus the distance, angle, dihedral parameters calculated from the first 4-ns MD simulations may not be the same after the subsequent simulations, which means that the restraint was not added at the equilibrium position (this can also happen quite often in other FEP calculations).

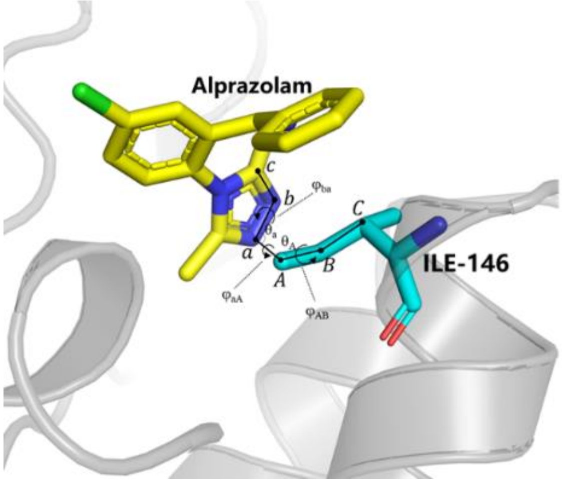

Figure S4. Manually selected restraint atoms.

For the above system, when we use two λ to add the restraint, the probability distribution of sampled ΔU cannot be fitted well by RED function (Figure S5). If we further examine the figure, the fitted function contains only Gaussian term with harmonic term to be zero. This happens because the added restraint is far from the equilibrium position and cannot be described by the RED function. For this system, the free energies calculated by using 12 λ and 2 λ are 13.0 kcal/mol and 16.4 kcal/mol, respectively. The difference is quite large, and thus the free energy cannot be calculated accurately by 2-λ. Thus, whether the sampled distribution can be fitted well by the RED function can be a criterion to determine if the restraint free energy can be calculated reasonably by the single-step perturbation.

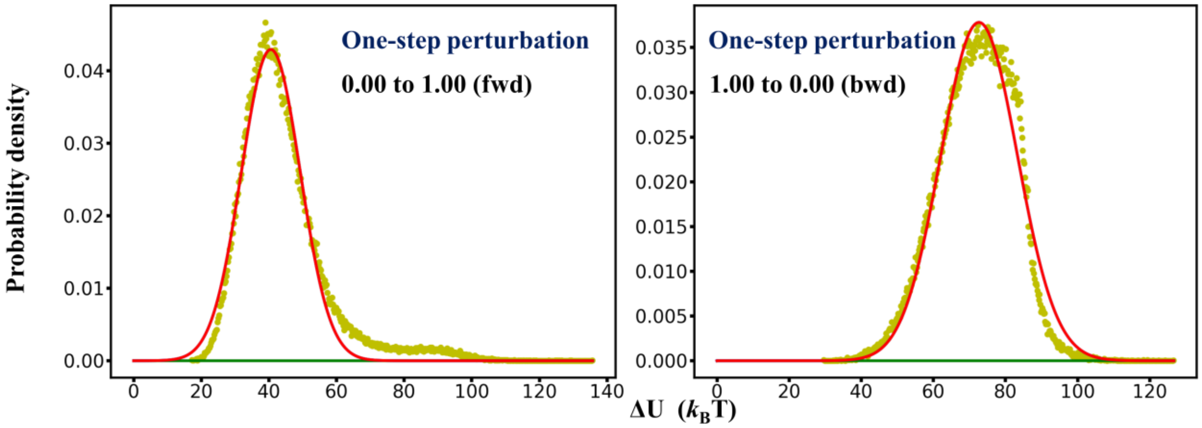

Figure S5. The probability distribution cannot be fitted well by the RED function if the restraint was not added at the equilibrium (or near-equilibrium) position.

Section S6. FEP calculations can be accelerated by >3-fold without losing the accuracy with appropriate selection of λ values

In order to further accelerate the calculation, we studied the effect of decreasing the number of λ values for both charge and vdW interactions decoupling by using 4 different receptor-ligand systems. The corresponding crystal structures (PDB codes: 3SVG, 3U5L, 3U5J, and 4HBV) were used in order to make sure that the binding modes between the receptors and their ligands are reasonable. For all the 4 crystal structures, ΔA^{RL} was first calculated by using 42 λ (12 λ for restraint, 10 λ for charge, 20 λ for vdW) as described in the recently published reports,(18, 19) and then the number of λ values were decreased for the decoupling of both the charge and vdW interactions. As seen in Table S3, by using 16 λ values in total (2 λ for restraint with fitting by the RED function, 5 λ for charge, and 9 λ for vdW), the energy calculation results are quite close to the corresponding results calculated by using 42 λ values for all the 4 systems tested (all the energy differences are within 1 kcal/mol). Compared with the 42 λ, the FEP-ABFE calculations using just 16 λ values need only 38% computational resources, and the calculations are accelerated by ~3-fold.

Table S3. The details of free energy changes for the complex in the annihilation for the traditional 42-window scheme and the 16-window scheme.[a]

λ [b]	3SVG		3U5L		3U5J		4HBV	
	$\Delta A^{RL}_{42\,\lambda}$ [c]	$\Delta A^{RL}_{16\,\lambda}$ [d]	$\Delta A^{RL}_{42\,\lambda}$	$\Delta A^{RL}_{16\,\lambda}$	$\Delta A^{RL}_{42\,\lambda}$	$\Delta A^{RL}_{16\,\lambda}$	$\Delta A^{RL}_{42\,\lambda}$	$\Delta A^{RL}_{16\,\lambda}$
(0.0, 0.0, 0.0)	0.000	0.000	0.000	0.000	0.000	0.000	0.000	0.000
(0.01, 0.0, 0.0)	0.239	-	0.016	-	0.029	-	0.030	-
(0.025,0.0,0.0)	0.394	-	0.055	-	0.067	-	0.085	-
(0.05, 0.0, 0.0)	0.539	-	0.117	-	0.121	-	0.165	-
(0.075,0.0,0.0)	0.697	-	0.154	-	0.165	-	0.219	-
(0.10, 0.0, 0.0)	0.914	-	0.202	-	0.204	-	0.271	-
(0.15, 0.0, 0.0)	1.365	-	0.290	-	0.277	-	0.366	-
(0.20, 0.0, 0.0)	1.681	-	0.346	-	0.369	-	0.449	-
(0.30, 0.0, 0.0)	2.086	-	0.476	-	0.528	-	0.592	-
(0.50, 0.0, 0.0)	2.736	-	0.699	-	0.723	-	0.833	-
(0.75, 0.0, 0.0)	3.416	-	0.863	-	0.924	-	1.082	-
(1.0, 0.0, 0.0)	3.992	3.503	1.002	0.797	1.082	1.104	1.294	1.351
(1.0, 0.1, 0.0)	6.963	-	3.123	-	3.184	-	3.715	-
(1.0, 0.2, 0.0)	9.391	9.390	5.077	4.872	5.122	5.141	5.815	5.420
(1.0, 0.3, 0.0)	11.611	-	6.874	-	6.881	-	7.613	-
(1.0, 0.4, 0.0)	13.466	13.252	8.456	8.253	8.429	8.436	9.259	9.001
(1.0, 0.5, 0.0)	14.830	-	9.817	-	9.821	-	10.667	-
(1.0, 0.6, 0.0)	15.896	15.600	10.999	10.935	10.978	10.964	11.613	11.288
(1.0, 0.7, 0.0)	16.669	-	12.003	-	11.931	-	12.253	-
(1.0, 0.8, 0.0)	17.236	16.888	12.758	12.671	12.568	12.814	12.692	12.641
(1.0, 0.9, 0.0)	17.631	-	13.285	-	13.012	-	12.902	-
(1.0, 1.0, 0.0)	17.857	17.459	13.662	13.535	13.526	13.519	12.985	13.206
(1.0, 1.0, 0.05)	19.052	-	15.025	-	14.877	-	13.992	-
(1.0, 1.0, 0.10)	20.241	19.867	16.387	16.286	16.205	16.064	14.995	15.197
(1.0, 1.0, 0.15)	21.395	-	17.738	-	17.503	-	15.979	-
(1.0, 1.0, 0.20)	22.505	22.206	19.070	18.981	18.737	18.579	16.977	17.238
(1.0, 1.0, 0.25)	23.599	-	20.381	-	19.928	-	17.978	-
(1.0, 1.0, 0.30)	24.631	24.448	21.657	21.554	21.107	20.923	18.969	19.265
(1.0, 1.0, 0.35)	25.617	-	22.914	-	22.247	-	19.939	-
(1.0, 1.0, 0.40)	26.622	-	24.170	-	23.323	-	20.889	-
(1.0, 1.0, 0.45)	27.580	-	25.419	-	24.365	-	21.847	-
(1.0, 1.0, 0.50)	28.516	28.478	26.652	26.620	25.433	25.260	22.825	23.244

(1.0, 1.0, 0.55)	29.379	-	27.876	-	26.487	-	23.790	-
(1.0, 1.0, 0.60)	30.118	-	28.984	-	27.458	-	24.713	-
(1.0, 1.0, 0.65)	30.660	-	30.000	-	28.361	-	25.501	-
(1.0, 1.0, 0.70)	31.043	31.015	30.883	30.937	28.974	28.870	25.961	26.490
(1.0, 1.0, 0.75)	31.292	-	31.599	-	29.356	-	26.174	-
(1.0, 1.0, 0.80)	31.108	31.127	32.084	32.108	29.559	29.722	26.115	26.429
(1.0, 1.0, 0.85)	30.272	-	31.945	-	29.208	-	25.673	-
(1.0, 1.0, 0.90)	29.402	29.738	31.368	31.081	28.490	29.323	25.189	25.547
(1.0, 1.0, 0.95)	28.986	29.362	31.069	30.555	28.098	28.918	24.969	25.369
(1.0, 1.0, 1.0)	29.156	29.520	31.237	30.613	28.206	29.008	25.128	25.508

[a] All the free energy differences are calculated by the BAR method. The unit of free energy differences is kcal/mol.

[b] In the lambda arrays, the first value refers to the restraint-lambdas and the second and third value represents the value of coul-lambdas and vdw-lambdas.

[c] $\Delta A_{42\,\lambda}^{RL}$ refers to the free energy difference between the initial state of perturbation and the specific alchemical state defined by the relative lambda arrays, whose alchemical pathway is defined by 42 windows.

[d] $\Delta A_{16\,\lambda}^{RL}$ refers to the free energy difference between the initial state of perturbation and the specific alchemical state defined by the relative lambda arrays, whose alchemical pathway is defined by 16 windows.

Section S7. Accuracy of the FEP-ABFE calculations based on the test results for the 28 receptor-ligand systems

The accuracy of the accelerated 16-λ FEP-ABFE calculation was first tested against 3 targets with 28 receptor-ligand systems. One of the targets was BRD4, which was also the focus of a study of the FEP-ABFE method using 42 λ values reported previously by Aldeghi *et al.*,(18, 19) and since the FEP-ABFE calculations show systematic bias when the net charge of a ligand is not 0, all 8 neutral ligands from the work of Aldeghi *et al*. were used in this study.(18) In addition to the target BRD4, the other two targets were HIV-1 protease (with 10 neutral ligands) and human factor Xa (with 10 neutral ligands). All the binding modes for the 28 test systems were obtained from the protein data bank (www.rcsb.org), which ensured the correctness of the initial structures.

As shown in detail in Figures S7-S9 and Table S4, the ligands were quite diverse in terms of the following features: the molecular weights ranging from 241 to 662 Da; number of atoms ranging from 22 to 89; number of rotatable bonds ranging from 0 to 21; number of hydrogen bond acceptors ranging from 1 to 7; number of hydrogen bond donors ranging from 0 to 6; and the calculated log*P* ranging from 1.17 to 4.85. The FEP-ABFE calculation results for all the 28 systems are summarized in Table S5, and the linear regression statistics for the calculated and experimental results are given in Figure S6. For the HIV-1 protease and human factor Xa, most of the predicted binding free energy (ΔG_{pred}) values are consistent with the corresponding experimental binding free energy (ΔG_{exp}) values, with an average prediction error being less than 2.0 kcal/mol. For BRD4, although the calculations showed some systematic error, with all the calculated results shifting in the negative direction, the calculated results still showed a good linear correlation with the experimental results. For comparison, the commonly used binding free energy calculation methods MM-PBSA (molecular mechanics Poisson-Boltzmann surface area) and MM-GBSA (molecular mechanics generalized-Born surface area) were also used to calculate the binding free energies for all the 28 receptor-ligand systems. The MM/GBSA and MM/PBSA calculations were carried out with the single-trajectory protocol. The gas-phase energies were calculated by using the AMBER FF14SB force

field which is the same one used in the MD simulations. In the MM/GBSA, Onufriev's GB model (33) was used for GB calculation, and LCPO algorithm was used (34) to calculate the nonpolar desolvation free energy with $G_{np} = 0.005 \times \Delta SASA$. In the MM/PBSA calculations, the radii optimized by Tan and Luo(35) was used by molsurf (36) to calculate the SASA. The nonpolar desolvation free energy was calculated based on G_{np} = 0.00542 (kcal/mol/Å^2) × ΔSASA (Å^2) + 0.9200 (kcal/mol). The dielectric constant value we used for the solute was set to 1 and dielectric constant value we used for the surrounding solvent was set to 80 in both PB and GB calculations. The entropy contributions were ignored in this study since the normal mode calculations are quite time-consuming. The detailed results obtained from the MM/PBSA and MM/GBSA calculations are given in Table S6 and Figure S6.

As seen in Figure S6, most of the results from the MM-PBSA and MM-GBSA calculations showed very poor correlation (or no significant correction) with the experimental data, with R^2 = 0.000 to 0.136 for the MM-PBSA results and R^2 = 0.150 to 0.366 for the MM-GBSA results for the three targets. In comparison, R^2 = 0.642 to 0.915 for the FEP-ABFE results, as seen in Figure S6. So, the FEP-ABFE results for all the three targets are markedly better than the corresponding MM-PBSA and MM-GBSA results. The test results based on the 28 ligands with diverse chemical scaffolds suggest that the accelerated FEP-ABFE algorithm can achieve a remarkable accuracy, which encouraged us to perform the FEP-ABFE prediction-based practical virtual screening to identify SARS-CoV-2 M^{pro} inhibitors for drug repurposing.

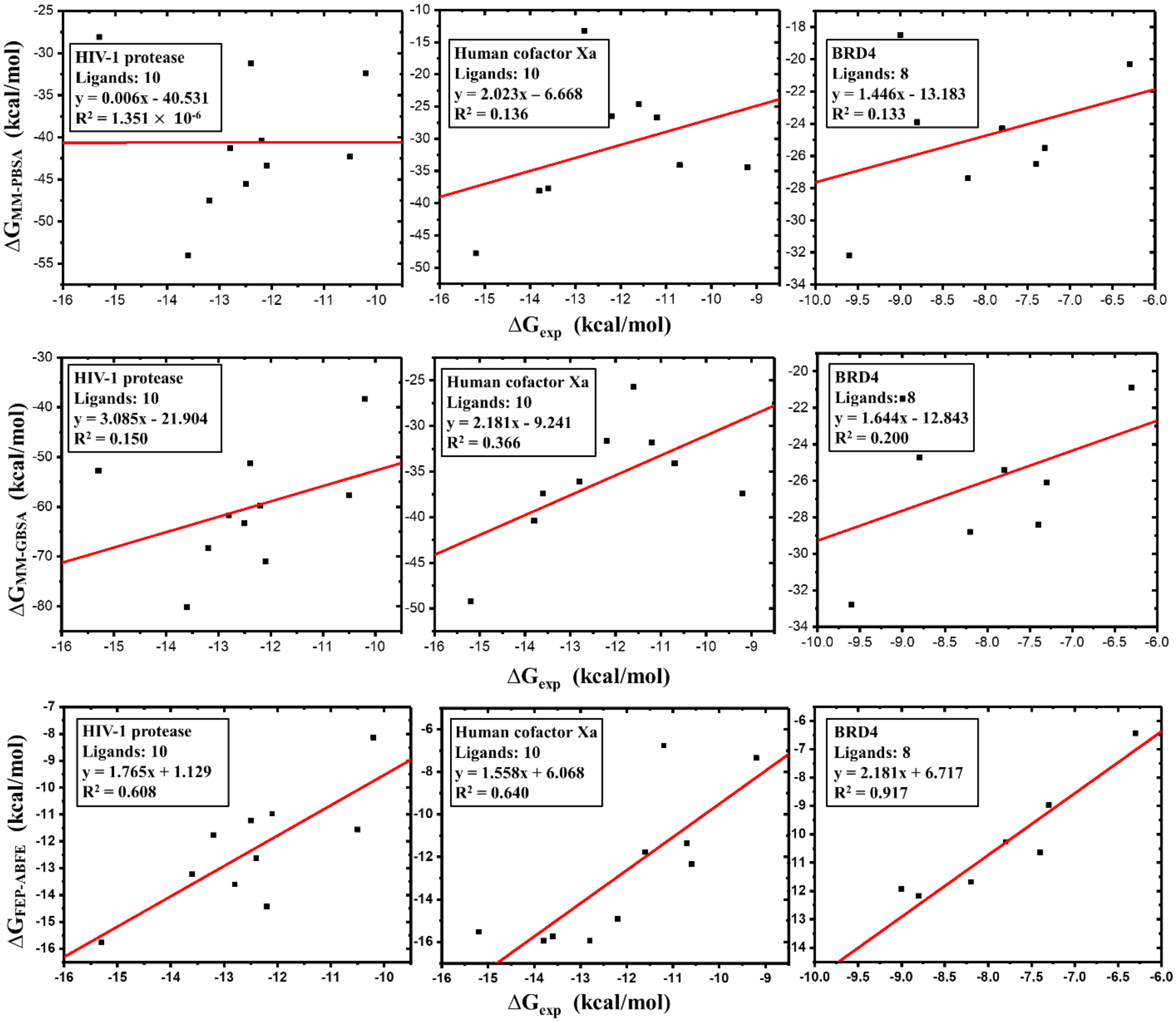

Figure S6. The regression models between experimental ΔG_{exp} and predicted $\Delta G_{MM\text{-}PBSA}$, $\Delta G_{MM\text{-}GBSA}$, and $\Delta G_{FEP\text{-}ABFE}$ values for the three targets and 28 ligands.

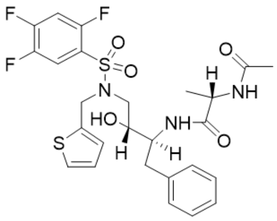

2QHY

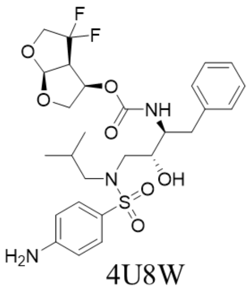

4U8W

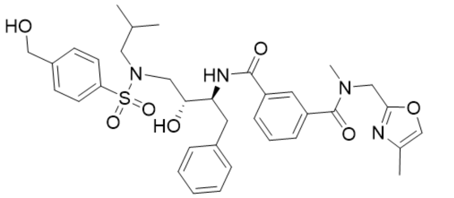

5UPZ

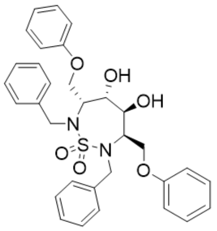

1AJV

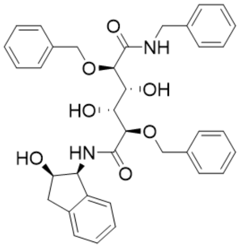

1D4H

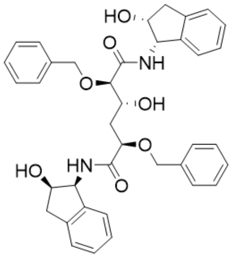

1D4I

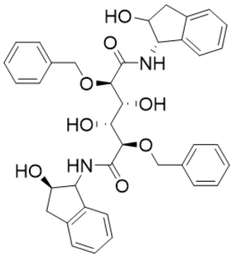

1EBY

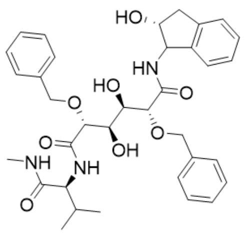

1EBZ

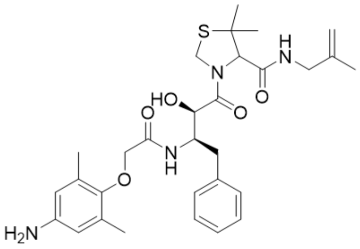

3A2O

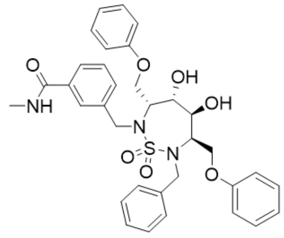

1G2K

HIV-1 protease

Figure S7. Molecular structures of the tested HIV-1 protease inhibitors

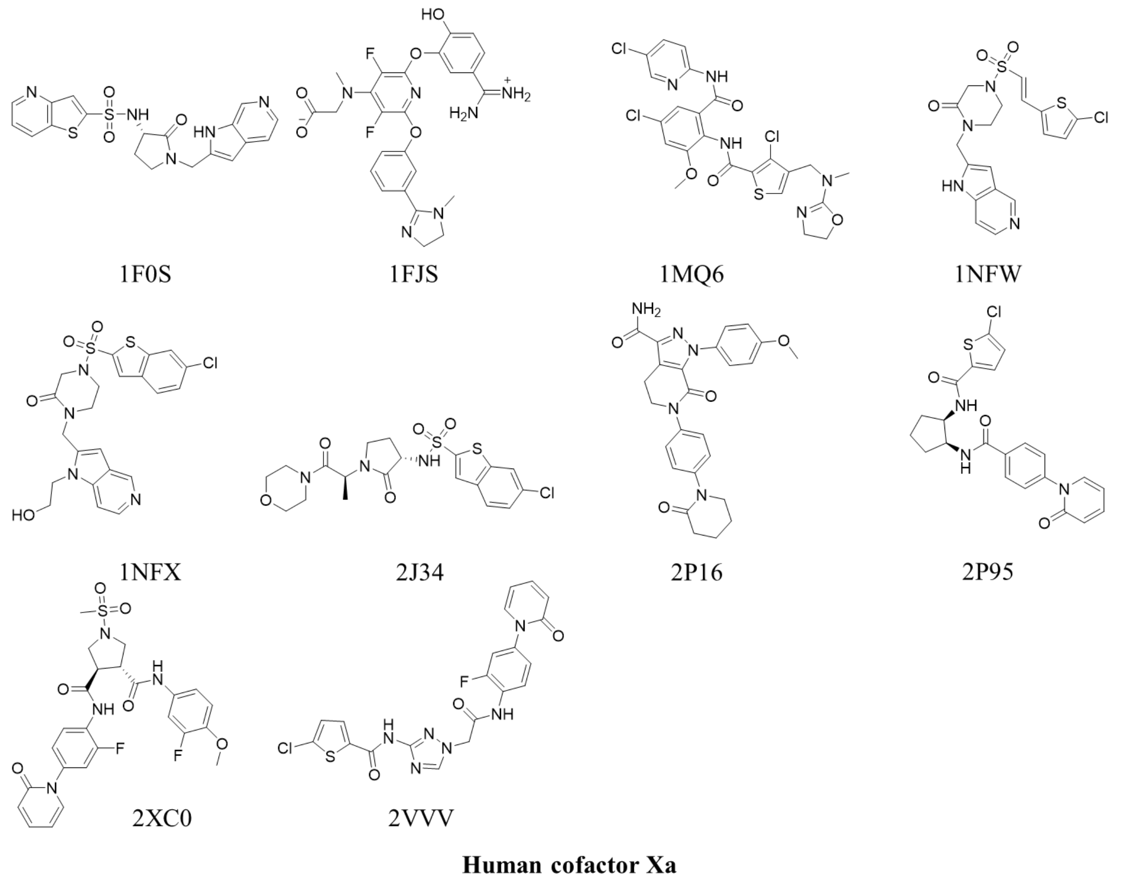

Figure S8. Molecular structures of the tested human cofactor Xa inhibitors

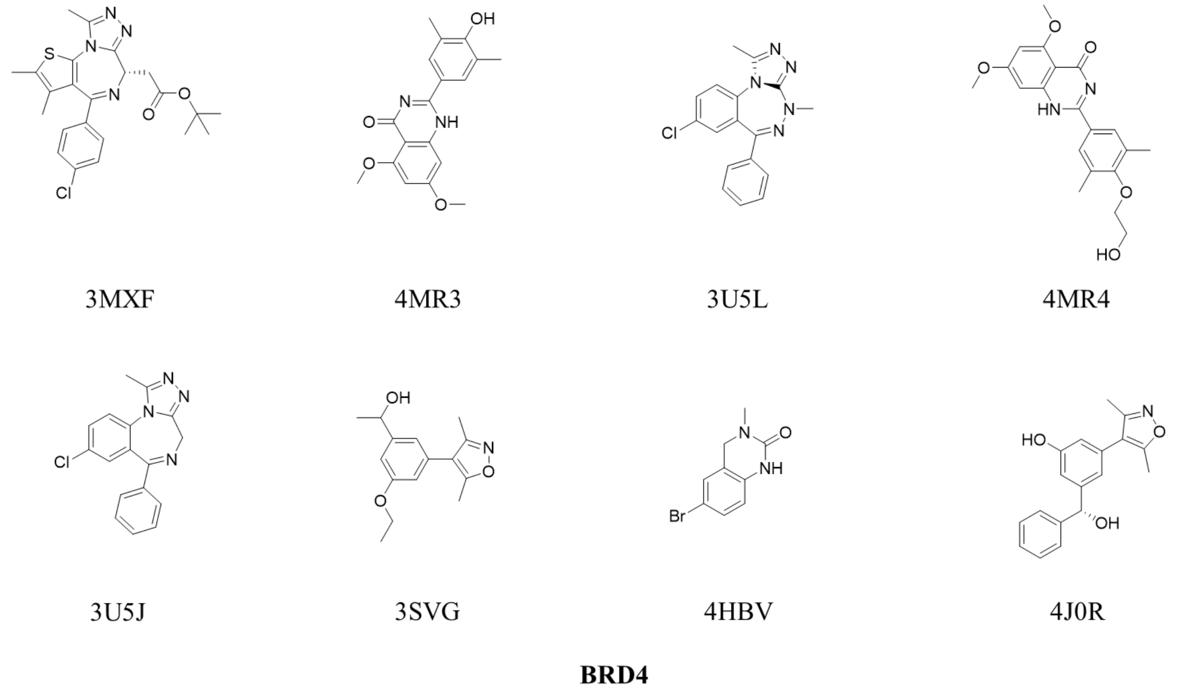

Figure S9. Molecular structures of the tested BRD4 inhibitors.

Table S4. Physical chemical properties of the 28 tested ligands. PDB refers the PDB code with bound ligands. MW is the molecular weight in Daltons; Netchg is the net charge; NROT is the number of rotatable bonds; HBA is the number of hydrogen bond acceptors; HBD is the number of hydrogen bond donors; cLogP is the calculated octanol/water partition coefficient (computed with XLOGP3). All the properties are gained from the PDBbind-CN Database.

PDB	MW(Da)	No. Atoms	Netchg	NROT	HBA	HBD	cLogP
2QHY	583.643	67	0	15	5	3	3.05
4U8W	583.645	75	0	14	4	3	3.25
5UPZ	662.796	89	0	19	7	3	3.70
1AJV	574.687	75	0	12	4	2	4.42
1D4H	610.696	83	0	19	5	5	3.43
1D4I	636.284	87	0	18	5	5	3.86
1EBY	652.733	88	0	19	6	6	3.44
1EBZ	633.731	89	0	21	6	6	2.65
3A2O	568.727	80	0	15	4	4	3.97
1G2K	631.738	82	0	14	5	3	3.69
1F0S	427.500	46	0	5	5	2	1.54
1FJS	526.500	63	0	10	4	1	4.76
1MQ6	568.860	56	0	10	3	2	4.66
1NFW	436.936	45	0	5	4	1	2.06
1NFX	505.010	54	0	7	5	1	2.14
2J34	471.978	52	0	6	4	1	2.12
2P16	459.497	59	0	5	4	1	2.24
2P95	441.931	50	0	7	3	2	4.15
2XC0	546.543	62	0	9	5	2	1.17
2VVV	472.880	46	0	8	5	2	3.28
3MXF	456.988	56	0	5	3	0	4.85
4MR3	326.347	42	0	4	2	2	2.67
3U5L	323.780	37	0	1	3	0	4.57
4MR4	370.399	49	0	7	2	2	2.31
3U5J	308.765	35	0	1	2	0	3.23
3SVG	261.321	38	0	4	4	1	2.21
4HBV	241.085	22	0	0	1	1	1.37
4J0R	295.332	39	0	5	3	2	3.25

Table S5. Summary of the FEP free energy calculation results (kcal/mol) for the three important drug targets including HIV-1 protease, human factor Xa, and BRD4.

Target	PDB	ΔG_{exp}	ΔG_{cal}	$\Delta G_{cal} - \Delta G_{exp}$
HIV-1 protease	2QHY	-10.2	-8.7±0.3	1.5
	4U8W	-15.3	-15.3±0.3	0.0
	5UPZ	-12.5	-11.0±0.4	1.5
	1AJV	-10.5	-11.8±0.4	-1.3
	1D4H	-13.6	-13.2±0.3	0.4
	1D4I	-12.1	-10.5±0.5	1.6
	1EBY	-13.2	-12.3±0.4	0.9
	1EBZ	-12.8	-13.5±0.4	-0.7
	3A2O	-12.4	-12.6±0.4	-0.2
	1G2K	-12.2	-13.4±0.4	-1.2
Human factor Xa	1F0S	-10.6	-10.2±0.2	0.4
	1FJS	-13.6	-15.8±0.8	-2.2
	1MQ6	-15.2	-14.8±0.2	0.4
	1NFW	-12.2	-14.3±0.2	-2.1
	1NFX	-11.6	-12.1±0.3	-0.5
	2J34	-10.7	-11.0±0.2	-0.3
	2P16	-13.8	-15.9±0.2	-2.1
	2P95	-12.8	-15.9±0.4	-3.1
	2XC0	-9.2	-7.0±0.3	2.2
	2VVV	-11.2	-6.4±0.3	4.8
BRD4	3MXF	-9.6	-14.5±0.2	-4.9
	4MR3	-9.0	-11.7±0.1	-2.7
	3U5L	-8.2	-11.6±0.1	-3.4
	4MR4	-7.8	-10.4±0.2	-2.6
	3U5J	-7.4	-10.8±0.1	-3.4
	3SVG	-7.3	-9.1±0.1	-1.8
	4HBV	-6.3	-6.8±0.1	-0.5
	4J0R	-8.8	-11.9±0.1	-3.1

ΔG_{exp} and ΔG_{cal} are the experimental binding free energy and FEP-calculated binding free energy, respectively.

Table S6. Summary of the MM-PBSA and MM-GBSA calculation results (kcal/mol) for the HIV-1 protease, human factor Xa, and BRD4 inhibitors. Eight nanoseconds MD simulations were performed for each receptor-ligand system, and MM-PBSA and MM-GBSA were calculated based on 100 snapshots extracted from the trajectories of the last 1-ns MD simulations (with an interval of 10 ps).

Target	PDB	ΔG_{exp}	ΔG_{MM_PBSA}	ΔG_{MM_GBSA}
HIV-1 protease	2QHY	-10.2	-32.4±4.2	-38.3±3.4
	4U8W	-15.3	-28.1±5.7	-52.7±4.2
	5UPZ	-12.5	-45.5±6.0	-63.3±3.6
	1AJV	-10.5	-42.3±4.6	-57.7±3.8
	1D4H	-13.6	-54.0±5.3	-80.1±4.6
	1D4I	-12.1	-43.4±5.9	-71.0±4.7
	1EBY	-13.2	-47.5±5.7	-68.3±4.4
	1EBZ	-12.8	-41.3±5.5	-61.7±4.3
	3A2O	-12.4	-31.2±6.4	-51.2±4.0
	1G2K	-12.2	-40.4±7.5	-59.7±3.9
Human factor Xa	1F0S	-10.6	-22.5±3.5	-25.4±2.6
	1FJS	-13.6	-37.7±8.1	-37.4±6.8
	1MQ6	-15.2	-47.8±3.2	-49.2±2.9
	1NFW	-12.2	-26.5±3.0	-31.6±2.4
	1NFX	-11.6	-24.6±3.1	-25.7±2.2
	2J34	-10.7	-34.1±3.1	-34.1±2.7
	2P16	-13.8	-38.1±3.4	-40.4±2.7
	2P95	-12.8	-13.2±8.3	-36.1±2.4
	2XC0	-9.2	-34.4±4.2	-37.4±4.6
	2VVV	-11.2	-26.7±3.1	-31.8±2.0
BRD4	3MXF	-9.6	-32.2±2.5	-32.8±1.8
	4MR3	-9.0	-18.5±2.9	-21.5±1.9
	3U5L	-8.2	-27.4±2.6	-28.8±2.3
	4MR4	-7.8	-24.3±2.6	-25.4±2.2
	3U5J	-7.4	-26.5±2.2	-28.4±1.9
	3SVG	-7.3	-25.5±2.5	-26.1±2.3
	4HBV	-6.3	-20.3±2.4	-20.9±1.7
	4J0R	-8.8	-23.9±2.2	-24.7±2.0

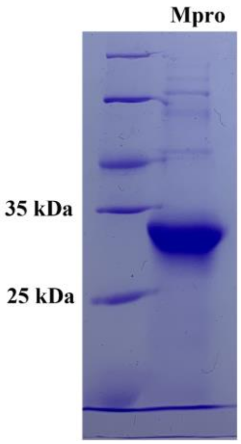

Figure S10. M^{pro} protein analyzed by SDS-PAGE.

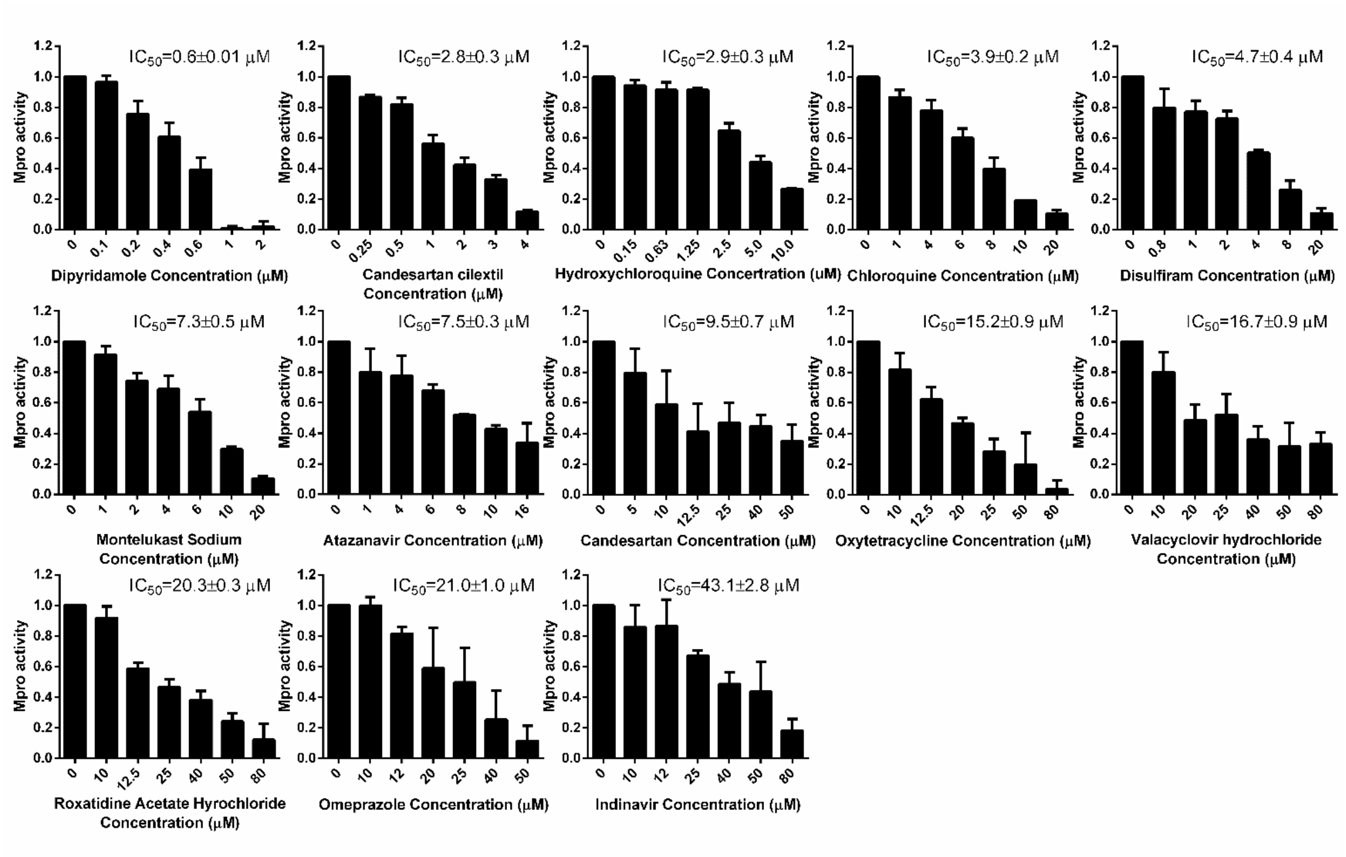

Figure S11. The inhibitory curves for the most potent M^{pro} inhibitors.

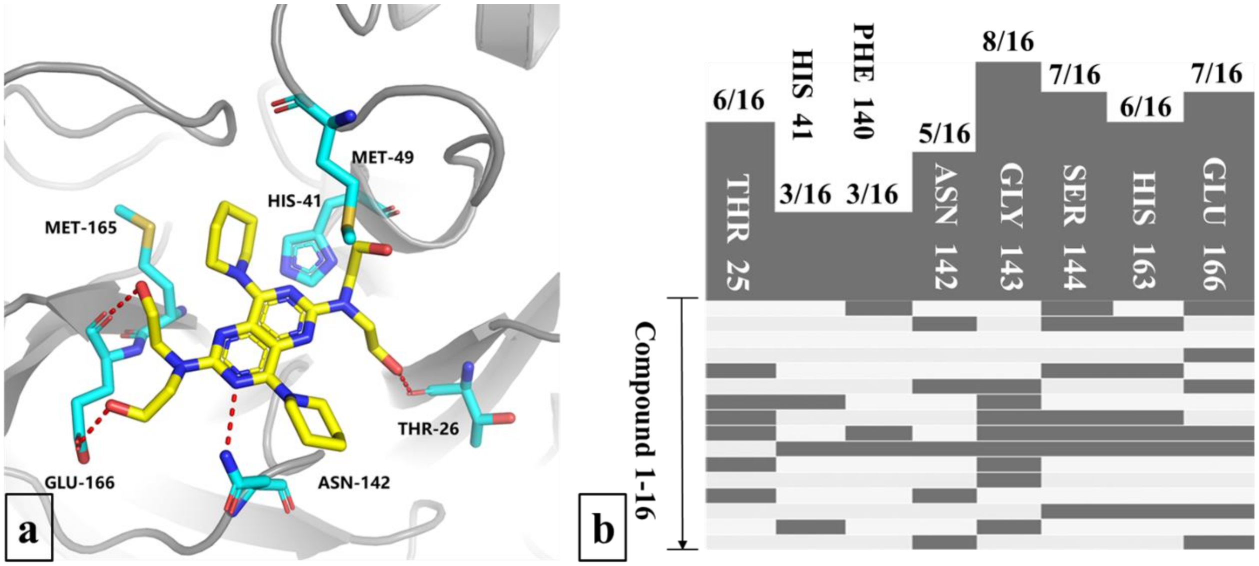

Figure S12. (a) Binding mode of M^{pro} with dipyridamole after the MD simulations. Dipyridamole is shown in yellow in the stick model, the key amino-acid residues of M^{pro} are shown in cyan in the stick model, and the hydrogen bonds are shown as red dashed lines. (b) The protein-ligand interaction fingerprint (PLIF) of the 16 potent inhibitors and M^{pro}.

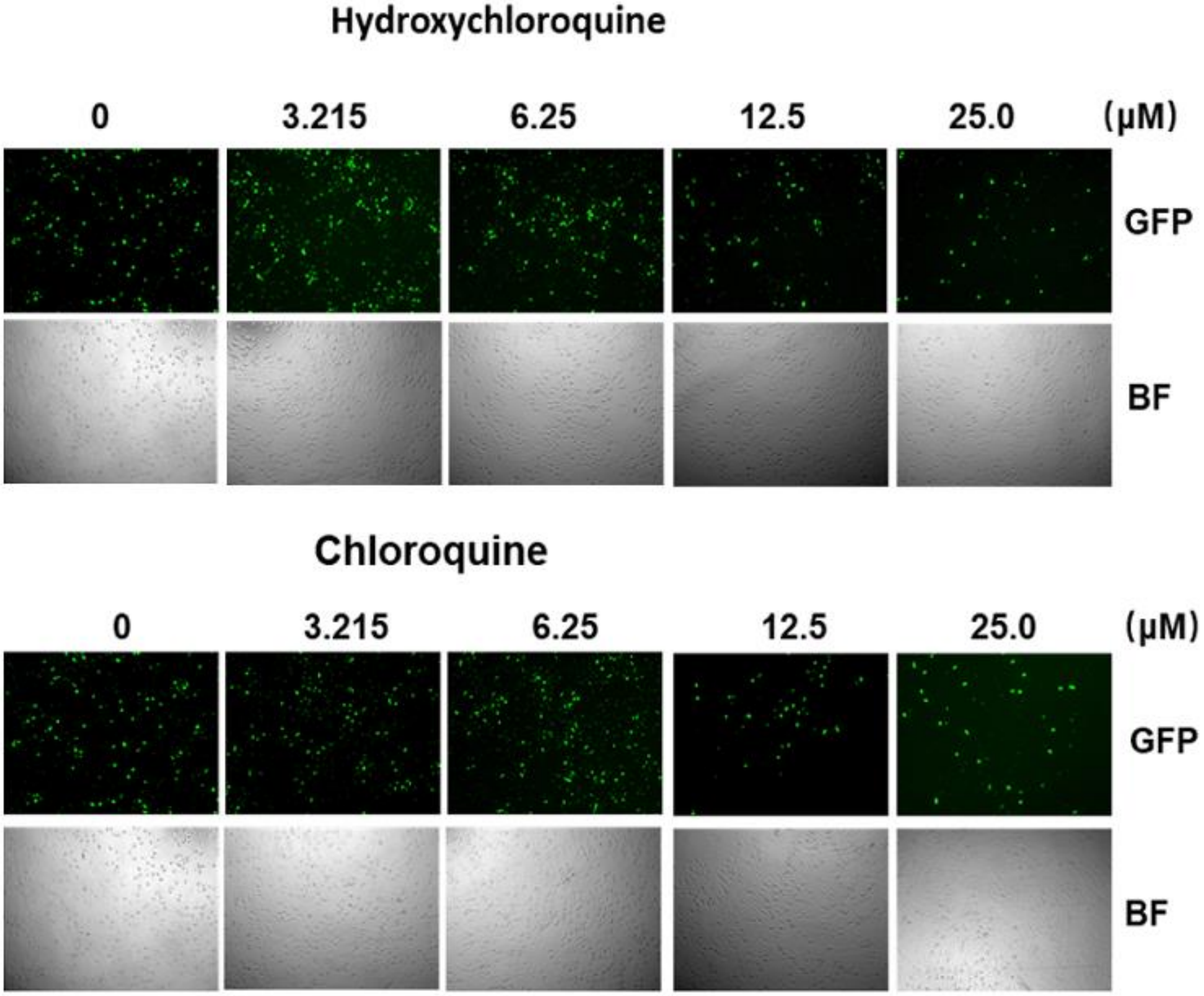

Figure S13. Chloroquine and its derivates do not inhibit VSV infection at low concentration. African green monkey kidney cell line VERO was infected with recombinant vesicular stomatitis virus, in which a reporter gene green fluorescence protein (GFP) was inserted into viral genome, at MOI (multiplicity of infection) 0.01. And then cells were treated with indicated chemicals at different concentration immediately. Ten hours post infection, viral infection was monitored under a microscope. Green fluorescence indicated infected cells. BF, bright field.

References for SI

1. M. Wang *et al.*, Remdesivir and chloroquine effectively inhibit the recently emerged novel coronavirus (2019-nCoV) in vitro. *Cell Res.* **30**, 269-271 (2020).
2. Z. Xu *et al.*, Nelfinavir is active against SARS-CoV-2 in Vero E6 cells. *ChemRxiv*, DOI: 10.26434/chemrxiv.12039888.v1 (2020).

3. L. Dong, S. Hu, J. Gao, Discovering drugs to treat coronavirus disease 2019 (COVID-19). *Drug Discov. Ther.* **14**, 58-60 (2020).
4. Z. Jin *et al.*, Structure of Mpro from SARS-CoV-2 and discovery of its inhibitors. *Nature* **582**, 289-293 (2020).
5. H. Li, A. D. Robertson, J. H. Jensen, Very fast empirical prediction and rationalization of protein pKa values. *Proteins: Struct., Funct., Bioinf.* **61**, 704-721 (2005).
6. D. C. Bas, D. M. Rogers, J. H. Jensen, Very fast prediction and rationalization of pKa values for protein–ligand complexes. *Proteins: Struct., Funct., Bioinf.* **73**, 765-783 (2008).
7. C. R. Søndergaard, M. H. Olsson, M. Rostkowski, J. H. Jensen, Improved treatment of ligands and coupling effects in empirical calculation and rationalization of p K a values. *J. Chem. Theory Comput.* **7**, 2284-2295 (2011).
8. M. H. Olsson, C. R. Søndergaard, M. Rostkowski, J. H. Jensen, PROPKA3: consistent treatment of internal and surface residues in empirical p K a predictions. *J. Chem. Theory Comput.* **7**, 525-537 (2011).
9. T. A. Halgren *et al.*, Glide: a new approach for rapid, accurate docking and scoring. 2. Enrichment factors in database screening. *J. Med. Chem.* **47**, 1750-1759 (2004).
10. G. L. Warren *et al.*, A critical assessment of docking programs and scoring functions. *J. Med. Chem.* **49**, 5912-5931 (2006).
11. B. Hess, C. Kutzner, D. Van Der Spoel, E. Lindahl, GROMACS 4: algorithms for highly efficient, load-balanced, and scalable molecular simulation. *J. Chem. Theory Comput.* **4**, 435-447 (2008).
12. S. Pronk *et al.*, GROMACS 4.5: a high-throughput and highly parallel open source molecular simulation toolkit. *Bioinformatics* **29**, 845-854 (2013).
13. J. Wang, R. M. Wolf, J. W. Caldwell, P. A. Kollman, D. A. Case, Development and testing of a general amber force field. *J. Comput. Chem.* **25**, 1157-1174 (2004).
14. M. Frisch *et al.*, Gaussian 03, revision C. 02; Gaussian, Inc.: Wallingford, CT, 2004. (2013).
15. D. A. Case *et al.*, The FF14SB force field. *Amber* **14**, 29-31 (2014).
16. W. L. Jorgensen, J. Chandrasekhar, J. D. Madura, R. W. Impey, M. L. Klein, Comparison of simple potential functions for simulating liquid water. *J. Chem. Phys.* **79**, 926-935 (1983).
17. S. Boresch, F. Tettinger, M. Leitgeb, M. Karplus, Absolute binding free energies: a quantitative approach for their calculation. *J. Phys. Chem. B* **107**, 9535-9551 (2003).
18. M. Aldeghi, A. Heifetz, M. J. Bodkin, S. Knapp, P. C. Biggin, Accurate calculation of the absolute free energy of binding for drug molecules. *Chem. Sci.* **7**, 207-218 (2016).

19. M. Aldeghi, A. Heifetz, M. J. Bodkin, S. Knapp, P. C. Biggin, Predictions of ligand selectivity from absolute binding free energy calculations. *J. Am. Chem. Soc.* **139**, 946-957 (2017).
20. G. Hummer, L. R. Pratt, A. E. García, Multistate Gaussian model for electrostatic solvation free energies. *J. Am. Chem. Soc.* **119**, 8523-8527 (1997).
21. Z. Li *et al.*, Absolute binding free energy calculation and design of a subnanomolar inhibitor of phosphodiesterase-10. *J. Med. Chem.* **62**, 2099-2111 (2019).
22. G. J. Rocklin, D. L. Mobley, K. A. Dill, P. H. Hünenberger, Calculating the binding free energies of charged species based on explicit-solvent simulations employing lattice-sum methods: An accurate correction scheme for electrostatic finite-size effects. *J. Chem. Phys.* **139**, 11B606_601 (2013).
23. W. F. Van Gunsteren, H. J. Berendsen, A leap-frog algorithm for stochastic dynamics. *Mol. Simul.* **1**, 173-185 (1988).
24. N. Goga, A. Rzepiela, A. De Vries, S. Marrink, H. Berendsen, Efficient algorithms for Langevin and DPD dynamics. *J. Chem. Theory Comput.* **8**, 3637-3649 (2012).
25. M. Parrinello, A. Rahman, Polymorphic transitions in single crystals: A new molecular dynamics method. *J. Appl. Phys.* **52**, 7182-7190 (1981).
26. B. Hess, H. Bekker, H. J. Berendsen, J. G. Fraaije, LINCS: a linear constraint solver for molecular simulations. *J. Comput. Chem.* **18**, 1463-1472 (1997).
27. U. Essmann *et al.*, A smooth particle mesh Ewald method. *J. Chem. Phys.* **103**, 8577-8593 (1995).
28. C. H. Bennett, Efficient estimation of free energy differences from Monte Carlo data. *J. Comput. Phys.* **22**, 245-268 (1976).
29. M. R. Shirts, J. D. Chodera, Statistically optimal analysis of samples from multiple equilibrium states. *J. Chem. Phys.* **129**, 124105 (2008).
30. J. Wang, Y. Deng, B. Roux, Absolute binding free energy calculations using molecular dynamics simulations with restraining potentials. *Biophys. J.* **91**, 2798-2814 (2006).
31. H. Fujitani *et al.*, Direct calculation of the binding free energies of FKBP ligands. *J. Chem. Phys.* **123**, 084108 (2005).
32. M. Lapelosa, E. Gallicchio, R. M. Levy, Conformational transitions and convergence of absolute binding free energy calculations. *J. Chem. Theory Comput.* **8**, 47-60 (2012).
33. A. Onufriev, D. Bashford, D. A. Case, Exploring protein native states and large-scale conformational changes with a modified generalized born model. *Proteins: Struct., Funct., Bioinf.* **55**, 383-394 (2004).

34. J. Weiser, P. S. Shenkin, W. C. Still, Approximate atomic surfaces from linear combinations of pairwise overlaps (LCPO). *J. Comput. Chem.* **20**, 217-230 (1999).
35. C. Tan, L. Yang, R. Luo, How well does Poisson− Boltzmann implicit solvent agree with explicit solvent? A quantitative analysis. *J. Phys. Chem. B* **110**, 18680-18687 (2006).
36. M. L. Connolly, Analytical molecular surface calculation. *J. Appl. Crystallogr.* **16**, 548-558 (1983).
